# GradHC: highly reliable gradual hash-based clustering for DNA storage systems

**DOI:** 10.1093/bioinformatics/btae274

**Published:** 2024-04-22

**Authors:** Dvir Ben Shabat, Adar Hadad, Avital Boruchovsky, Eitan Yaakobi

**Affiliations:** Department of Computer Science, Technion, Haifa 320003, Israel; Department of Computer Science, Technion, Haifa 320003, Israel; Department of Computer Science, Technion, Haifa 320003, Israel; Department of Computer Science, Technion, Haifa 320003, Israel

## Abstract

**Motivation:**

As data storage challenges grow and existing technologies approach their limits, synthetic DNA emerges as a promising storage solution due to its remarkable density and durability advantages. While cost remains a concern, emerging sequencing and synthetic technologies aim to mitigate it, yet introduce challenges such as errors in the storage and retrieval process. One crucial task in a DNA storage system is clustering numerous DNA reads into groups that represent the original input strands.

**Results:**

In this paper, we review different methods for evaluating clustering algorithms and introduce a novel clustering algorithm for DNA storage systems, named Gradual Hash-based clustering (GradHC). The primary strength of GradHC lies in its capability to cluster with excellent accuracy various types of designs, including varying strand lengths, cluster sizes (including extremely small clusters), and different error ranges. Benchmark analysis demonstrates that GradHC is significantly more stable and robust than other clustering algorithms previously proposed for DNA storage, while also producing highly reliable clustering results.

**Availability and implementation:**

https://github.com/bensdvir/GradHC.

## 1 Introduction

DNA storage is an emerging technology that promises to revolutionize the way we store and preserve digital data (Goldman *et al.* 2013, Grass *et al.* 2015, Ceze *et al.* 2019). By encoding data as synthetic DNA, it becomes possible to store vast amounts of information in a very compact form that can last for thousands of years, without the need for electricity or any other external power source (Grass *et al.* 2015, Organick *et al.* 2018). Moreover, DNA is much more durable than other storage media, such as hard drives, which can degrade or fail over time, and it is also very energy efficient (Goldman *et al.* 2013, Kosuri and Church 2014, Grass *et al.* 2015). However, the cost of DNA storage is still a significant barrier to its widespread adoption. Although the price of DNA synthesis has been declining rapidly in recent years, it is still much higher than other storage technologies (Goldman *et al.* 2013, Kosuri and Church 2014, Grass *et al.* 2015, Organick *et al.* 2018, Ceze *et al.* 2019). Moreover, the process of storing and retrieving DNA data is complex and time-consuming, involving several steps that require specialized expertise and equipment.

To use DNA as a storage medium, the first step is to map binary data onto DNA strands by replacing every two bits with their corresponding DNA nucleotide [adenine (A), thymine (T), guanine (G), and cytosine (C)]. Because of limitations in synthesis technology, the data is divided into short strands no longer than a few hundred nucleotides (Kosuri and Church 2014). Next, these DNA strands can be encoded using error-correcting codes or other encoding techniques. These DNA strands are then synthesized, yielding hundreds to thousands of noisy copies of each original strand which are stored in a container. When data needs to be retrieved, a small sample of DNA strands is taken from the container and amplified using a technique called polymerase chain reaction (PCR). After amplification using PCR, each DNA strand is sequenced to determine its DNA symbols. However, the synthesis, PCR, and sequencing steps are all susceptible to errors, including insertions, deletions, and substitutions, which can result in errors in the stored data. Each error type occurs with a different probability, and the overall error rates are mainly influenced by the synthesis and sequencing technologies used (Grass *et al.* 2015, Erlich and Zielinski 2017, Organick *et al.* 2018, Ceze *et al.* 2019). The outcome of the sequencing step is an unordered set of thousands to millions of DNA strands, consisting of multiple noisy copies of the original DNA strand. Before the decoding process, a *clustering algorithm*, which is the focus of this work, must be used to group the noisy copies together so that each cluster contains copies of the same original DNA strand. After clustering, each cluster is reconstructed using a *reconstruction algorithm* to achieve an approximation of the original DNA strand (Batu *et al.* 2004, PS18, Viswanathan and Swaminathan 2008, Srinivasavaradhan *et al.* 2021, Sabary *et al.* 2024). Finally, once all the DNA strands have been retrieved, the coding scheme is used to decode them, and the reverse mapping is applied to obtain the original binary data.

In this work, we aim to address the problem of clustering in DNA-based storage systems. We will review all existing clustering algorithms and identify their strengths and weaknesses. In addition, we will propose the GradHC algorithm, a novel clustering approach specifically designed for DNA storage systems. To evaluate the effectiveness of our proposed method, we will conduct benchmarking experiments on previous experiments and simulated datasets and compare the results against those of state-of-the-art clustering algorithms for DNA storage.

## 2 DNA clustering

### 2.1 Problem definition

As presented in the Introduction, a key aspect of every DNA data-storage system is the *clustering* phase. When reading the strands after the sequencing process, one gets multiple noisy copies from every input strand. The number of copies varies from a few to thousands and depends on the sequencing technology, the number of sampling cycles, and the PCR process. Before applying a *reconstruction* algorithm to get the original input, one must cluster the strands together, in a way that with high probability every cluster consists of noisy copies of the same input strand. Formally, a clustering *C* of a finite set $S\subseteq \Sigma^{*}$, is any partition of *S* into nonempty subsets, when Σ={A,C,G,T} and $S=\{s_{1},s_{2},\ldots ,s_{n}\}$ is the set of all DNA reads gathered in the sequencing process. One must note that clustering DNA strands differs from traditional clustering problems in the way that the clustering algorithm is not given with essential parameter settings such as density threshold or cluster size distribution. In addition, computing a distance matrix between every pair of input strands, often necessary for clustering algorithms, is unfeasible due to the large-scale input size of DNA-based storage systems. The edit distance (a string metric allows for quantifying how similar two strings are to each other. It is measured by counting the minimum number of operations required to transform one string into the other) algorithm, commonly used for string similarity, has a quadratic time complexity of $O(l^{2})$, where *l* represents strand length, resulting in exponential computation costs with larger inputs. Thus, making the clustering task of DNA-storage system data a much more difficult problem. Moreover, in DNA storage systems, strands are not ordered in the memory, making it challenging to determine their storage order. The solution is to use indices that are stored as part of the strand to indicate its location relative to other strands. However, a naive algorithm that solely uses the index to cluster the strands may not work well due to potential errors in the index field.

### 2.2 Related work

Various DNA clustering algorithms have been developed, primarily from the fields of bioinformatics and metagenomics. Notable methods include UCLUST (Edgar 2010) and CD-HIT (Fu *et al.* 2012), which employ greedy algorithms and Needleman-Wunsch global alignment for sequence similarity. DNACLUST (Ghodsi *et al.* 2011) also uses a greedy approach with fast sequence alignment techniques. MeShClust v1.0 (James *et al.* 2018) utilizes the mean shift algorithm, while MeShClust v3.0 (Girgis 2022) combines the mean shift algorithm with alignment-free identity scores. MetaDEC (Bao *et al.* 2022) applies deep unsupervised learning, while MMseqs2 (Steinegger and Söding 2018) uses a graph-based approach.

A common weakness in many DNA-clustering algorithms, including those mentioned, is their use of a single sequence identity threshold. Still, selecting the right threshold is complex, relying on domain expertise, especially challenging in DNA storage systems.

ALFATClust (Chiu and Ong 2022) dynamically determines threshold values based on cluster separation and intra-cluster similarity. SEED (Bao *et al.* 2011) uses hashing techniques with interval seeding. It is important to note that all of these algorithms are primarily designed for genomic or metagenomic data, which feature longer DNA strands (tens of thousands of nucleotides) compared to the shorter strands (50–200 nucleotides) in DNA storage systems. Hence, these algorithms are not suitable for such a system.

Starcode (Zorita *et al.* 2015) is designed for very short DNA barcodes, while DBSCAN (Ester *et al.* 1996) is one of the earliest clustering algorithms but impractical for handling hundreds of thousands of strands. Shinkar *et al.* (2022) introduced an index-based clustering using a novel coding scheme. However, their correctness relies on the strong assumption that most strands within a cluster are derived from the same original strand.

Another approach in Rashtchian *et al.* (2017) introduced a distributed algorithm for clustering billions of reads within an hour, using a hashing scheme to estimate the edit distance. Antkowiak *et al.* (2020) presented an LSH-based clustering algorithm for a DNA storage system. Clover (Qu *et al.* 2022) is a DNA storage clustering algorithm with linear computational complexity and low memory usage since it avoids Levenshtein distance computations.

More information regarding experiments and details about the methods used in the previous works mentioned above can be found in the Supplementary Material.

### 2.3 Performance validation

Cluster validation is a computational task, used for the evaluation of clustering algorithms in many fields, including bioinformatics, machine learning, data compression, and DNA storage. Currently, a wide variety of clustering evaluation metrics exist in these different fields. The reason for such a variety is due to the multiple aspects of correctness by which the accuracy and goodness of clustering algorithms can be evaluated, and the different requirements on the clustering results in each field. In most common metrics, clustering results are evaluated based on “ground truth” labels—i.e. perfect clustering labels. The perfect clustering of a DNA storage system is given by clustering every noisy copy with its original strand from which it was created. When simulated datasets are used, the perfect clustering is known in advance, since for each noisy copy generated, its original strand is known during the dataset creation process. Computing the perfect clustering of a wet experiment dataset is a more complex task. The best approach for solving this challenge is through an iterative greedy method: each read is associated with the original strand to which it has the best edit-distance score among all original strands. In this section, we will present two popular metrics that will be used to compare the results of the different clustering algorithms in our work.

1. **Threat Score (TS)**—the threat score [also known as critical success index (CSI) or Jaccard index (Bao *et al.* 2011)] is a verification measure ranging from 0 to 1, used to quantify the similarity between two datasets. Given a clustering $\tilde{C}$ and a perfect clustering *C* of a finite set $S\subseteq \Sigma^{*}$, we define *TP*, *FP*, and *FN*, (the overall number of true-positives, false-positives, and false-negatives, respectively) by:
  $$\begin{array}{l} TP=\sum_{i=1}^{\left|C\right|}\sum_{s\in C_{i}}1\left\{s\in {\tilde{C}}_{\pi^{\prime }(i)}\right\},\;FN=\sum_{i=1}^{\left|C\right|}\sum_{s\in C_{i}}1\left\{s\notin {\tilde{C}}_{\pi^{\prime }(i)}\right\},\\ FP=\sum_{i=1}^{\left|\tilde{C}\right|}\sum_{s\in {\tilde{C}}_{i}}1\left\{s\notin C_{\pi^{\prime }(i)}\right\},\;TS=\frac{TP}{TP\;+\;FP\;+\;FN}, \end{array}$$
  where $\pi^{\prime }$ is an injective map (for the injective map $\pi^{\prime }$ it holds that: 1. If $\pi^{\prime }(i)>|\tilde{C}|$, then ${\tilde{C}}_{\pi^{\prime }(i)}=\emptyset$. 2. We also define $\pi^{\prime }(i)=j$ if $\pi^{\prime }(j)=i$, and if such a *j* does not exist, then $C_{\pi^{\prime }(i)}=\emptyset$. These definitions also apply to the injective map π)
  $$\begin{array}{c} \pi^{\prime }:\left\{1,2,\ldots ,|C|\right\}\to \left\{1,2,\ldots ,\max(|C|,|\tilde{C}|)\right\},s.t:\\ \pi^{\prime }=\underset{\pi }{\text{argmax}}\sum_{i=1}^{\left|C\right|}|{\tilde{C}}_{\pi (i)}\cap C_{i}|. \end{array}$$
2. **Accuracy(** γ **)**—This metric was first defined in Rashtchian *et al.* (2017). For two clusterings $C,\tilde{C}$ of finite set $S\subseteq \Sigma^{*}$, the Accuracy(γ) for 0.5 < γ ≤ 1 of $\tilde{C}$ with respect to *C*, is given by [we slightly revised the original definition for Accuracy(γ) as defined in Rashtchian et al. (2017). However, both definitions refer to the same evaluation metric]:
  $$A_{\gamma }(C,\tilde{C})=\underset{\pi }{\max}\frac{1}{\left|C\right|}\sum_{i=1}^{\left|C\right|}1\left\{{\tilde{C}}_{\pi (i)}\subseteq C_{i},\text{and}\;|{\tilde{C}}_{\pi (i)}|\;\geq \;\gamma |C_{i}|\right\},$$
  where the maximum is taken over all injective maps (for the injective map $\pi^{\prime }$ it holds that: 1. If $\pi^{\prime }(i)\;>\;|\tilde{C}|$, then ${\tilde{C}}_{\pi^{\prime }(i)}=\emptyset$. 2. We also define $\pi^{\prime }(i)=j$ if $\pi^{\prime }(j)=i$, and if such a *j* does not exist, then $C_{\pi^{\prime }(i)}=\emptyset$. These definitions also apply to the injective map π):
  $$\pi :\left\{1,2,\ldots ,|C|\right\}\to \left\{1,2,\ldots ,\max(|C|,|\tilde{C}|)\right\}.$$

The accuracy $A_{\gamma }(C,\tilde{C})$ measures the number of clusters in $\tilde{C}$ that overlap with some cluster in *C* in at least a γ-fraction of elements while containing no false-positives. It is important to note that the strict constraint for the resulting clusters to have zero false-positives, may not be necessarily needed in DNA storage systems. This follows since some reconstruction algorithms may overcome the presence of a small number of false-positives, if the cluster is sufficiently large. Nevertheless, it should be remembered that in DNA storage systems the sizes of the clusters vary and in many cases may be extremely small. Additionally, there is great significance in the evaluation of the clustering algorithm’s results, regardless of the performance quality of the reconstruction algorithm used.

We chose the metrics listed above, as we find them to be the most suitable and precise for the validation of clustering algorithms for DNA storage systems. A thorough justification for the selection of these metrics, along with a discussion of additional evolution methods can be found in the Supplementary Material.

## 3 The GradHC algorithm

### 3.1 Algorithm description

**Figure 1.**
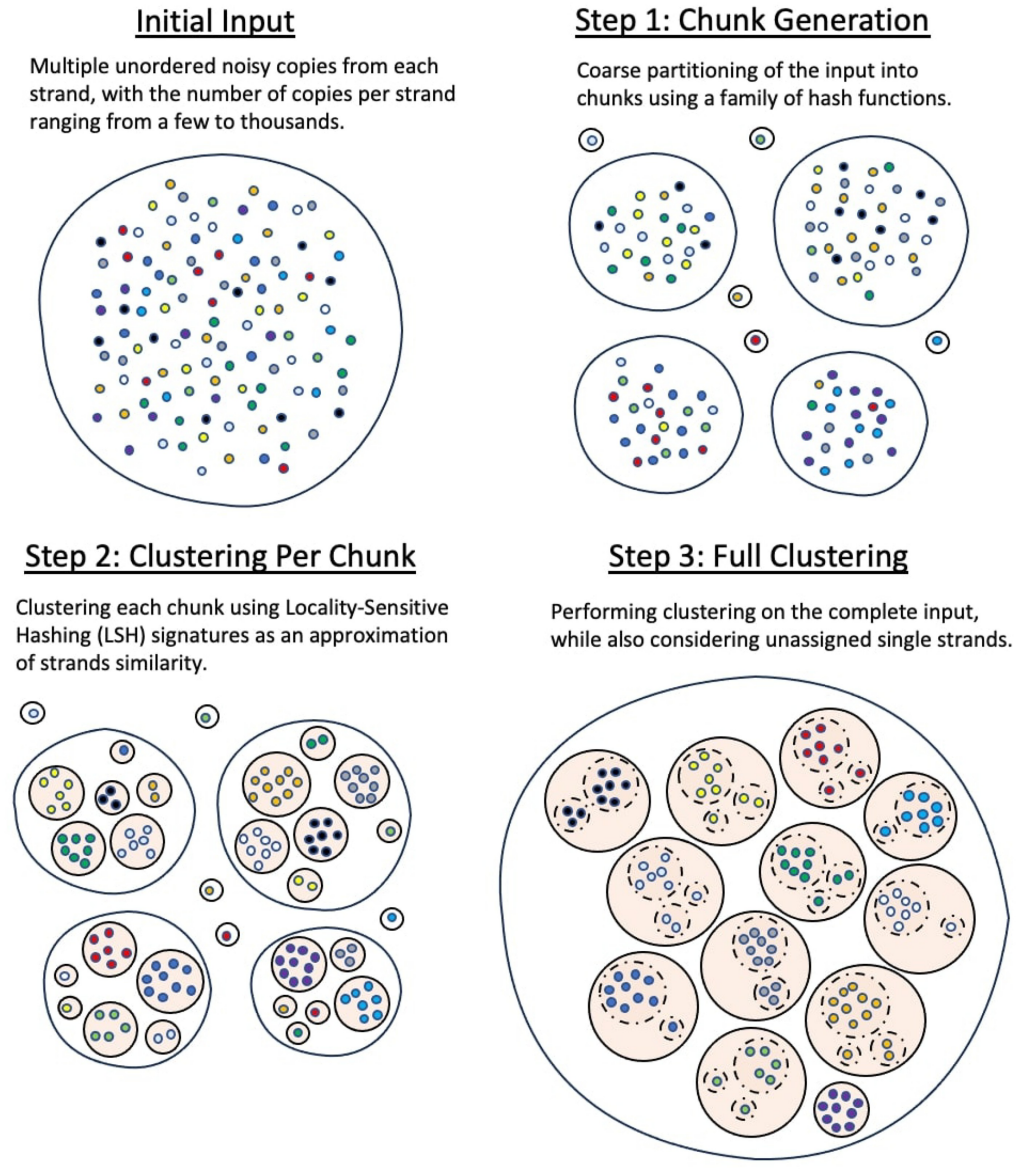
Schematic diagram of the GradHC model.

In this section, we present the *Gradual Hash-based Clustering* algorithm. Our algorithm was developed to deal with a wide range of error rates and draws inspiration from the clustering algorithm presented in Rashtchian *et al.* (2017) as well as the LSH-based clustering, suggested by Antkowiak *et al.* (2020). We designed an algorithm that can handle datasets with varying cluster sizes, from small (a few strands only) to large (hundreds to thousands of strands). Our clustering algorithm consists of three parts (see Fig. 1). First, we perform a primary step to obtain a coarse partition of the noisy copies with the aim of minimizing time costs afterward. Next, we execute the second step individually on each chunk of noisy copies generated in the previous step. Finally, a more computationally intensive third step is carried out, taking into account the entire input dataset.

#### 3.1.1 Step 1—Division into chunks

Algorithm 1 Chunks Generation

**Input:** *S-* a set of *n* noisy copies, w,t,mergers_ratio,p

**Output:** *C-* a partition of *S* into chunks

C←{{s},∀s∈S}

merges_count←0 , double_sig← True

finish← False

**repeat**

 sample $h_{y,t},h_{\hat{y},t}∼H_{w,t}$ //see below

 **for**$C_{i}\in C$**do**

  choose a representative $sr_{C_{i}}$

  **if**double_sig*is True* **then**

   $\mathrm{sig}(C_{i})\leftarrow (h_{y,t}(sr_{C_{i}}),h_{\hat{y},t}(sr_{C_{i}}))$

  **else**

   $\mathrm{sig}(C_{i})\leftarrow h_{y,t}(sr_{C_{i}})$

  **end**

 **end**

 **for**$C_{i},C_{j}$*s.t.* $\mathrm{sig}(C_{i})=\mathrm{sig}(C_{j})$**do**

  $C\leftarrow (C\setminus \{C_{i},C_{j}\})\cup \{C_{i}\cup C_{j}\}$

  merges_count←merges_count + 1

 **end**

 **if**$\frac{\mathrm{merges}\_\mathrm{count}}{n}\;\leq \;\mathrm{mergers}\_\mathrm{ratio}$*for p* times in a row **then**

  **if**double_sig*is False* **then**

   finish← True

  **else**

   double_sig← False

  **end**

 **end**

**until** *finish is True*;

return *C*

The algorithm begins by dividing the *n* noisy copies of *S* into initial clusters (chunks). The goal is to improve performance in the next steps of the algorithm, where the actual clustering process happens. The partitioning is supposed to be coarse and cheap. Let $x=x_{1}x_{2}\cdots x_{r}$ be a given strand, and let $y\in \Sigma^{w}$ be a random strand of length *w*, and *i* be the index of the first occurrence of *y* in *x*. We define $h_{y,t}(x)$ (we define $h_{y,t}(x)=\epsilon$ if there is no occurrence of *y* in *x*. In our algorithm, we consider the signatures $\mathrm{sig}(C_{i}),\mathrm{sig}(C_{j})$ as equal only if they do not contain ϵ at all) to be the substring $x_{i}x_{i+1}\cdots x_{r^{\prime }}$, where $r^{\prime }=\min(r,i\;+\;w\;+\;t\;-\;1)$.Similar to the definition in Rashtchian *et al.* (2017), the family of hash functions $H_{w,t}$ (parameterized by *w* and *t*), is given by: $H_{w,t}=\{h_{y,t}:\Sigma^{*}\to \Sigma^{w+t}\cup \{\epsilon \}\;|\;y\in \Sigma^{w}\}$. In the algorithm, we sample $h_{y,t}$ from $H_{w,t}$ by choosing a random strand $y\in \Sigma^{w}$. In this step, we make use of a skeleton (note that the revised definition for applying the hash scheme to *x* is mathematically equivalent to the one presented in Rashtchian *et al.* (2017)] of the algorithm suggested by Rashtchian *et al.* (2017), with several modifications. First, as accuracy is not a necessity, we refrain from computing edit distance. Second, in the first iterations, we use two signatures (generated by their proposed family of hash function $H_{w,t}$), and only after several iterations, the algorithm moves toward relying on a single signature. The motivation behind the changes was to keep the computation fast, resulting in a partition into chunks aimed to resemble the original clusters, making the next steps easier. An explanation of parameter selection for Step 1 can be found in the Supplementary Material.

Step 1 of the algorithm involves merging clusters of sequences with identical signatures. This merging technique results in a division of the input into smaller inputs (chunks), to be later clustered in Step 2. The generation of these chunks significantly contributes to the overall efficiency of the algorithm: In Steps 2 and 3, the algorithm merges clusters of sequence pairs, involving computationally intensive calculations. To optimize this process and avoid the examination of numerous amount of pairs in the original input, we utilize the chunk division method. By doing so, we reduce the overall number of pairs requiring analysis. It is essential to note that the chunk division is not arbitrary and sequences belonging to the same cluster are placed into the same chunk. This is because the division is determined by the similarity of the representatives of the clusters. These representatives are selected in direct relation to their scores, which are continually updated based on their participation in merges. As demonstrated in Rashtchian *et al.* (2017), the family of hash functions $H_{w,t}$ serves as an estimation of edit distance. Despite the high number of false-positives, this method is acceptable at this step, given the substantial clustering performed in Steps 2 and 3. Additionally, it is worth mentioning that the algorithm ensures cost-effectiveness in the division process, as there are no additional calculations on pairs with the same hash-based signature in this step.

#### 3.1.2 Step 2—Clustering per chunk

Algorithm 2 Clustering Per Chunk

**Input:** *C* - a clustering of *S* (chunks), $q,m,k,\theta_{\mathrm{high}},\theta_{\mathrm{low}},\mathrm{thres}$,

   max_iterations

**Output:** $\hat{C}$ - a new clustering of *S*

$\hat{C}\leftarrow \emptyset$

**for** $C^{\prime }\in C$ **do**

 $\tilde{C}\leftarrow \{\{s_{i}\},\forall s_{i}\in C^{\prime }\}$

 generate *m* permutations of $\left\{0,\ldots ,4^{q-1}\right\}$: $\Pi =\{\pi_{0},\ldots ,\pi_{m-1}\}$

 **for**$s_{i}$*in* $C^{\prime }$**do**

   $N_{s_{i}}\leftarrow \text{Create}\;\text{Numset}(s_{i})$

   $\mathrm{sigs}[s_{i}]\leftarrow \text{Create}\;\text{LSH}\;\text{signature}(N_{s_{i}},\Pi )$

 **end**

 **for** *j in* 0,…,max_iterations**do**

   randomly select $j_{0},\ldots ,j_{k-1}$ from {0,…,m−1}

   **for**$s_{i}$*in* $C^{\prime }$**do**

     $\mathrm{cur}\_\mathrm{sig}[s_{i}]$ = $\left(\mathrm{sigs}[s_{i}][j_{0}],\ldots ,\mathrm{sigs}[s_{i}][j_{k-1}]\right)$

  **end**

  **for**$s_{u},s_{v}$*s.t.* $\mathrm{cur}\_\mathrm{sig}[s_{u}]=\mathrm{cur}\_\mathrm{sig}[s_{v}]$**do**

    **if** *sørensen_dice(*$N_{s_{u}}$, $N_{s_{v}}$*)* $\geq \;\theta_{\text{high}}$***or****(sørensen_dice(*$N_{s_{u}}$, $N_{s_{v}}$*)* $\geq \;\theta_{\text{low}}$***and****edit_distance(*$s_{u}$, $s_{v}$*)* ≤*thres)* **then**

    $\tilde{C}\leftarrow (\tilde{C}\setminus \{{\tilde{C}}_{s_{u}},{\tilde{C}}_{s_{v}}\})\cup \{{\tilde{C}}_{s_{u}}\cup {\tilde{C}}_{s_{v}}\}$

   **end**

  **end**

 **end**

 $\hat{C}\leftarrow \hat{C}\cup \tilde{C}$

**end**

**return** $\hat{C}$

**Function** Create Numset(*s*)**:**

 base←{A:0,C:1,G:2,T:3}

 *numset* ←∅

 **for** *q-gram in s* **do**

  num=0

  **for** *i in* 0,…,q−1**do**

   $\mathrm{num}=\mathrm{num}\;+\;(4^{i}\cdot \mathrm{base}[s[i]]$)

  **end**

  numset=numset∪{num}

 **end**

 return *numset*

**End Function**

**Function** Create LSH Signature(N,Π)**:**

 $\vec{\mathrm{signature}}=\vec{0}$

 **for**$\pi_{i}\in \Pi =\{\pi_{0},\ldots ,\pi_{m-1}\}$**do**

  $\vec{\mathrm{signature}}[i]=\mathrm{MinHas}h_{\pi_{i}}(N)$ //see below

 **end**

 return *signature*

**End Function**

In the second step, the DNA sequences are treated as sets of *q-grams—*overlapping sub-strings of length *q*. By assigning each character from {A,G,C,T} a number, we obtain an integer in base 4, representing the *q-gram*.

##### 3.1.2.1 Fundamental ideas

Let $N_{s_{i}}$ be a set of numbers of a sequence $s_{i}$, and π be a permutation of all the possible integers allowed to appear in such set. The following serves as a *MinHash* signature of $s_{i}$:

$$\mathrm{MinHas}h_{\pi }(N_{s_{i}})=\min\{\pi [i]\;|\;i\in N_{s_{i}}\}.$$

As demonstrated in Leskovec *et al.* (2014), *MinHash* serves as a robust approximation to the Jaccard Coefficient, effectively estimating the similarity between two sets. Consequently, sequences sharing the same *MinHash* signature for a given permutation π are considered to have similar numsets $N_{s_{i}}$ and $N_{s_{j}}$, indicating a high probability of being similar. The combination of several *MinHash* signatures is referred to as an *LSH signature*, as introduced in Antkowiak *et al.* (2020). We denote the length of the signature (the number of *MinHash* signatures composing it) as *k*. Local-Sensitive Hashing (LSH) has proven to be a powerful technique, initially designed for approximate nearest neighbor search in high-dimensional spaces, as introduced by Har-Peled *et al.* (Indyk and Motwani 1998). Moreover, LSH has found valuable applications in computational biology, where it serves as an effective tool for estimating distance metrics between sequences (Marçais *et al.* 2019). Furthermore, it has been successfully employed in the computation of approximate solutions for all-pairs local alignment in large-scale sequence comparisons (Buhler 2001). Expanding on the versatility of LSH and its various applications mentioned earlier, we applied LSH signatures comparison to identify potentially similar pairs of sequences. Thus, we assert that sequences sharing identical LSH signatures may originate from the same input strand.

Another method we used to approximate the Normalized-Edit-Distance (minimum number of edit operations required to transform one sequence into another, normalized by the maximum possible edit distance between the sequences) (NED) between two sequences $s_{i},s_{j}$ is the Sørensen–Dice coefficient (Carass *et al.* 2020), which allows us to compare the similarity of $N_{s_{i}}\;\text{and}\;N_{s_{j}}$. For two vectors *X, Y*, the Sørensen–Dice coefficient is defined by:

$$\mathrm{DSC}=\frac{2|X\cap Y|}{\left|X|+|Y\right|}.$$

The algorithm requires the *LSH signatures* of sequences to be equal before setting them in the same cluster. We add another condition, as this approach results in many false-positives, and requires the Sørensen–Dice similarity to be above a certain threshold. Details and experimental analyses investigating LSH and Sørensen–Dice coefficient as proxies for NED are provided in the Supplementary Material.

Unlike the original work in Antkowiak *et al.* (2020), we improve performance by roughly dividing the input into chunks, as shown earlier in Step 1. This process reduces the total number of comparisons needed and minimizes the run time. Moreover, during the run of our algorithm, we also maintain a score for each sequence. The score is a counter, incremented each time a sequence is paired with another. Here, we assume that a sequence similar to many other sequences in its cluster (and as a result, has a high score), can serve as a good representative of its cluster in the next step.

At this step, an in-depth calculation is carried out for each chunk independently. Following Step 1, we obtain reduced input sets of noisy copies which are then clustered individually. The objective in this step is to conduct more profound calculations, as accuracy at this point becomes significantly crucial. As previously explained, the correctness of this step relies on the correlation between the *LSH signatures* and the Sørensen-Dice coefficient, to the NED of two sequences. In this step, we assume that a pair of sequences with relatively low NED values are highly likely to originate from the same original input strand. We emphasize the use of strict threshold values to avoid unnecessary merges. To achieve this, we employ $\theta_{\text{high}}$ and $\theta_{\text{low}}$ to denote two approximation ranges. When the sequence’s similarity falls within the threshold values, we directly calculate their edit distance to avoid missing any crucial merges. Due to the demanding criteria employed in this step, this step achieves its goal of clustering each chunk with relatively high accuracy. The final step of the algorithm involves merging clusters that may have been dispersed among different chunks.

##### 3.1.2.2 Parameter selection

A detailed explanation of algorithm parameter selection (*q*, *k*, and the number of iterations) can be found in the Supplementary Material.

#### 3.1.3 Step 3—Full clustering

Algorithm 3 Full Clustering

**Input:** *C-* a clustering of $S,k,m,\theta_{\text{high}},\theta_{\text{low}},\text{thres}$

**Output:** $\hat{C}$ - a new clustering of *S*

finish←False

**repeat**

 $r_{0}\leftarrow |\{C^{\prime },\forall C^{\prime }\in C\;s.t.\;|C^{\prime }|=1\}|$

 **for**$C_{i}\in C$**do**

  choose a representative $sr_{C_{i}}$

 **end**

 randomly select $j_{0},\ldots ,j_{k-1}$ from {0,…,m − 1}

 **for**$C_{i}\in C$**do**

  $\mathrm{cur}\_\mathrm{sig}[sr_{C_{i}}]$ = $\left(\mathrm{sigs}[sr_{C_{i}}][j_{0}],\ldots ,\mathrm{sigs}[sr_{C_{i}}][j_{k-1}]\right)$

 **end**

 **for**$sr_{C_{i}},sr_{C_{j}}$*s.t* $\mathrm{cur}\_\mathrm{sig}[sr_{C_{i}}]=\mathrm{cur}\_\mathrm{sig}[sr_{C_{j}}]$**do**

  **if** *sørensen_dice(*$N_{sr_{C_{i}}}$, $N_{sr_{C_{j}}}$*)* $\geq \;\theta_{\text{high}}$***or***

   *(sørensen_dice (*$N_{sr_{C_{i}}}$, $N_{sr_{C_{j}}}$*)* $\geq \;\theta_{\text{low}}$***and***

  *edit_distance (*$sr_{C_{i}}$, $sr_{C_{j}}$*)* ≤*thres)* **then**

   $\tilde{C}\leftarrow (\tilde{C}\setminus \{\tilde{C_{i}},\tilde{C_{j}}\})\cup \{\tilde{C_{i}}\cup \tilde{C_{j}}\}$

  **end**

 **end**

 $r_{1}\leftarrow |\{C^{\prime },\forall C^{\prime }\in C\;s.t.\;|C^{\prime }|=1\}|$

 **if**$\frac{r_{1}-r_{0}}{r_{1}}\;\leq \;0.5\%$*for 8 times in a row* **then**

  replace representatives

 **end**

 **if**$\frac{r_{1}-r_{0}}{r_{1}}\;\leq \;\sqrt[{5}]{n}$*for 4 times in a row* **then**

  finish←True

 **end**

**until** *finish is True*;

**return** $\hat{C}$

The final step is the most dominant step of our clustering process. This step includes iterating over all the clusters created, choosing representatives from each one, and then executing the flow from the previous step, now with respect to all the sequences without limiting to a certain chunk. This step has proven to be very essential, as it manages to efficiently and accurately handle the numerous single sequences (clusters of size 1) not yet merged into one of the other clusters. As mentioned before, the representatives are selected based on their scores. When the number of merges made during several consecutive iterations has decreased, the representatives are replaced. This follows the fact that other highly scored sequences in a cluster were proved to be helpful as well. The motivation for repeating the same process as in Step 2, but now with the whole input, is the need to merge clusters from different chunks, altogether with better handling of the singles. The choice of similarity threshold values for this step is detailed in the Supplementary Materials.

The two main goals of Step 3, as mentioned before, are to merge clusters dispersed across different chunks, and to handle the not yet merged single sequences. To achieve these goals, we apply a method similar to that used in Step 2, with two important modifications: (i) Clusters merges are based on the similarity of their representatives. After the previous step, we are relying on representative sequences proven to have high scores (since they participated in multiple merges before). We also update representatives to allow for a wider variety of merges that have not been considered in previous iterations. (ii) Lower similarity thresholds are applied due to the reliability of the representatives. Moreover, the adjusted thresholds enable finding potential candidate clusters for single sequences to be merged with, considering that these singles are likely to be much more erroneous.

### 3.2 Time complexity of the algorithm

The run time for our proposed algorithm is O(nl) in the worst case, where *n* is the number of input sequences, and *l* is the strands’ length. An extended runtime analysis of the algorithm can be found in the Supplementary Material.

## 4 Results

For the testing, a setup with the following specifications was used: 32-core Intel Xeon E5-2630 CPU, 128 GB RAM. Several datasets were tested, with the purpose of evaluating the performance of the algorithm on diverse datasets, with different characteristics. The datasets used differ in the following settings: the number of strands in the original design (equal to the number of clusters in the perfect clustering), the length of the original strands (*l*), the total number of noisy copies in the dataset (*n*), the sizes of the clusters, and the error rates of the technologies used. The datasets that were used consist of simulated datasets, and datasets from prior DNA storage studies (Grass *et al.* 2015, Erlich and Zielinski 2017, Organick *et al.* 2018, Srinivasavaradhan *et al.* 2021). The benchmark analysis was performed with the following clustering algorithms:

1. Rashtchian *et al.*’s clustering algorithm (Rashtchian *et al.* 2017), implemented [the algorithm’s code was independently reproduced by us, following the algorithm outlined in Rashtchian *et al.* (2017)] with 780 serial iterations.
2. The Clover clustering algorithm (Qu *et al.* 2022), with its suggested parameters: HorizontalDrift=3, VerticalDrift=3, and treeDepth=15.
3. The LSH-based clustering algorithm presented in Antkowiak *et al.* (2020), with its recommended settings. ($k=4,K_{\mathrm{LSH}}=3$).

Starcode Zorita *et al.* (2015) is considered to be one of the most appropriate clustering algorithms for DNA storage systems (Sankar *et al.* 2022). However, according to Qu *et al.* (2022), while Starcode is extremely fast, it fails to achieve high-quality clustering. Furthermore, Rashtchian *et al.* (2017) demonstrated that their clustering algorithm outperforms the Starcode algorithm, despite the fact that the latter was tested using various distance threshold parameters (d∈{2,4,6,8}). Therefore, we have chosen not to include Starcode in our benchmark.

As will be shown later (and will be further discussed in Section 5), the algorithm in Rashtchian *et al.* (2017) exhibits significant instability, as manifested in its correctness and running time. To illustrate this issue, we conducted multiple runs (10) of the algorithm on several datasets and analyzed the average results and standard deviation values of the metrics.

### 4.1 Experimental datasets

**Figure 2.**
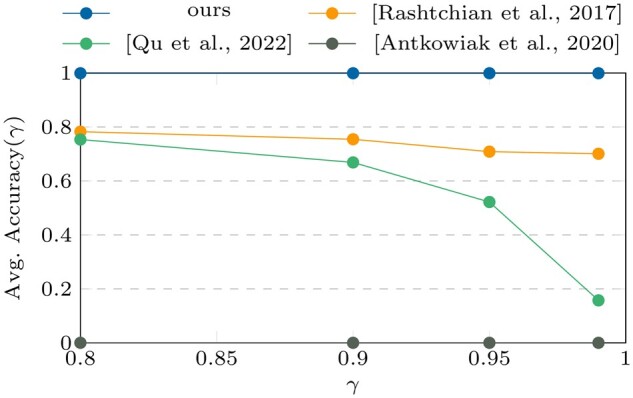
Avg. accuracy (γ) results on dataset I.

**Figure 3.**
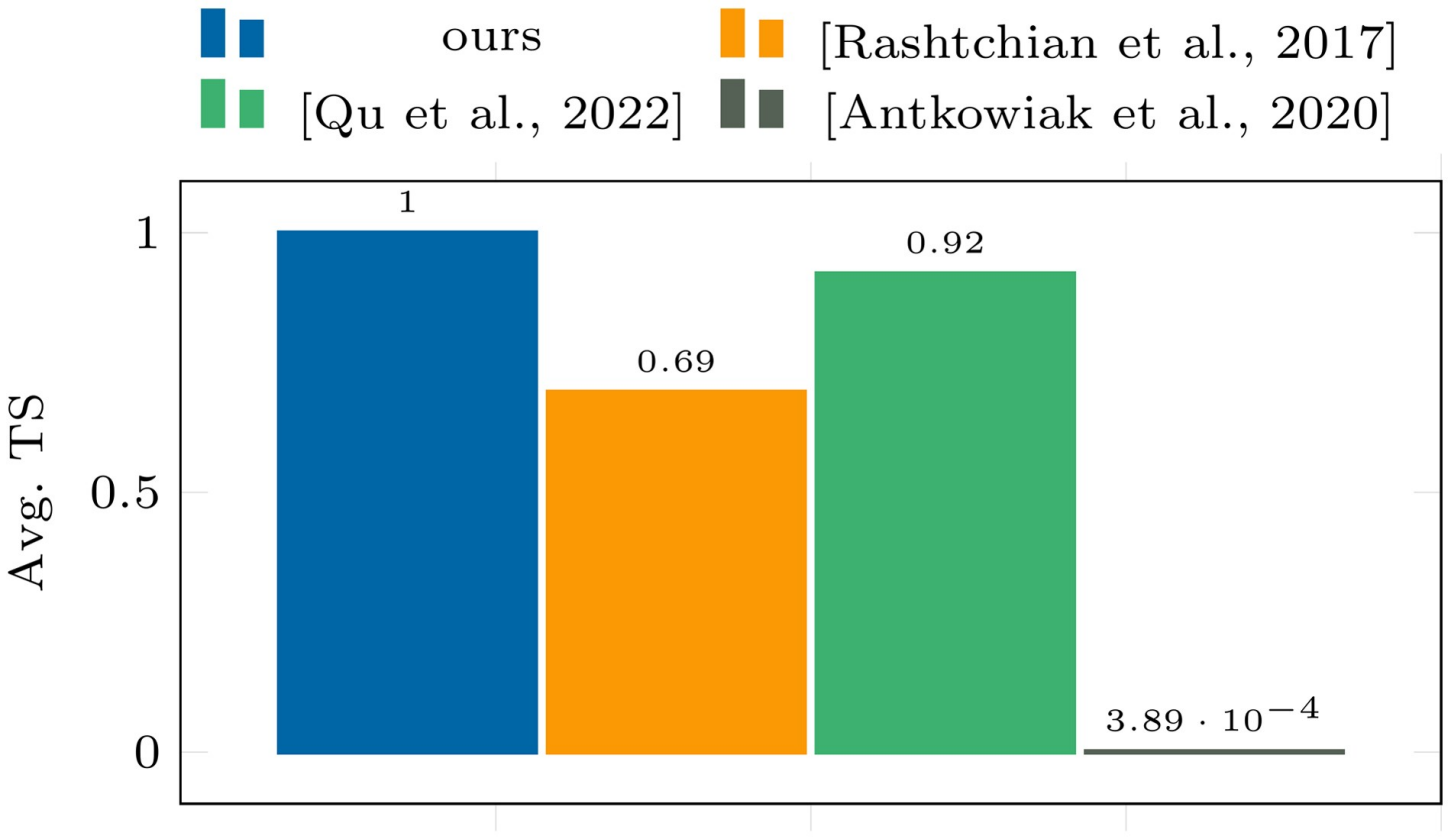
Avg. TS results on dataset I.

Our experimental datasets were collected from real DNA storage experiments performed in recent years. A detailed description of the datasets and their characteristics can be found in Table 1. The evolution of the algorithms on dataset I refers to the results of 10 runs of each algorithm on the dataset. In Figs 2 and 3, it can be clearly seen that our algorithm outperforms all other algorithms. The summary of the average results and the values of the standard deviations between all the runs appear in Tables 2 and 3.

**Table 1.** Properties of experimental datasets.

	Ref.	Data	Copies	Len.	Clust.	Err. (%)		
					Sizes	sub	ins	del
I	Grass *et al.* (2015)	4989	2 949 757	117	1–8863	0.331	0.0503	0.604
II	Erlich and Zielinski (2017)	72 000	13 332 276	152	1–683	0.968	0.0582	0.162
III	Organick *et al.* (2018)	596 499	11 378 301	110	1–87	0.254	0.0412	0.134
IV	Srinivasavaradhan *et al.* (2021)	9984	269 709	110	1–164	1.07	1.64	2.01

**Table 2.** Results summary of dataset I.

	Avg. Acc.	SD Acc.	Avg. TS	SD TS
	(γ=0.95)	(γ=0.95)		
Ours	**0.9991**	0.0006	**0.9994**	0.0007
Rashtchian *et al.* (2017)	0.7086	0.4696	0.6926	0.4723
Qu *et al.* (2022)	0.5219	0.0058	0.9208	0.0021
Antkowiak *et al.* (2020)	2.76$\;\cdot \;10^{-4}$	1.84$\;\cdot \;10^{-4}$	3.89$\;\cdot \;10^{-4}$	3.35$\;\cdot \;10^{-5}$

The bolded values highlight the best result among all algorithms in the benchmark.

**Table 3.** Runtime summary of dataset I.

	Avg. runtime (*s*)	SD Runtime (*s*)
Ours	984	22.33
Rashtchian *et al.* (2017)	5386	3779.52
Qu *et al.* (2022)	**13**	**0.28**
Antkowiak *et al.* (2020)	1177	5.62

According to the results presented in Figs 2 and 3, our algorithm outperforms all others on dataset I. Notably, Clover (Qu *et al.* 2022) achieves reasonably good results, considering its impressive running time. The clustering algorithm proposed by Rashtchian *et al.* (2017) exhibits instability, as reflected in the notably high values of Accuracy(γ) and TS standard deviation, as presented in Table 2. This instability is further evident in the extended running times detailed in Table 3. In practice, the algorithm introduced by Rashtchian *et al.* (2017) demonstrates several instances of high-quality results, both in terms of runtime and correctness, across some of the 10 runs. These results are comparable to or even better than those achieved by our algorithm. However, in some runs, it fails to converge, resulting in low Accuracy(γ) and TS values, along with extended running times. Tables 2 and 3 show that the remaining algorithms exhibited good stability with consistently low standard deviation values.

The results of the algorithms on dataset II closely resemble those of dataset I. Further results discussion together with detailed results for all algorithms on dataset II can be found in the Supplementary Material.

**Figure 4.**
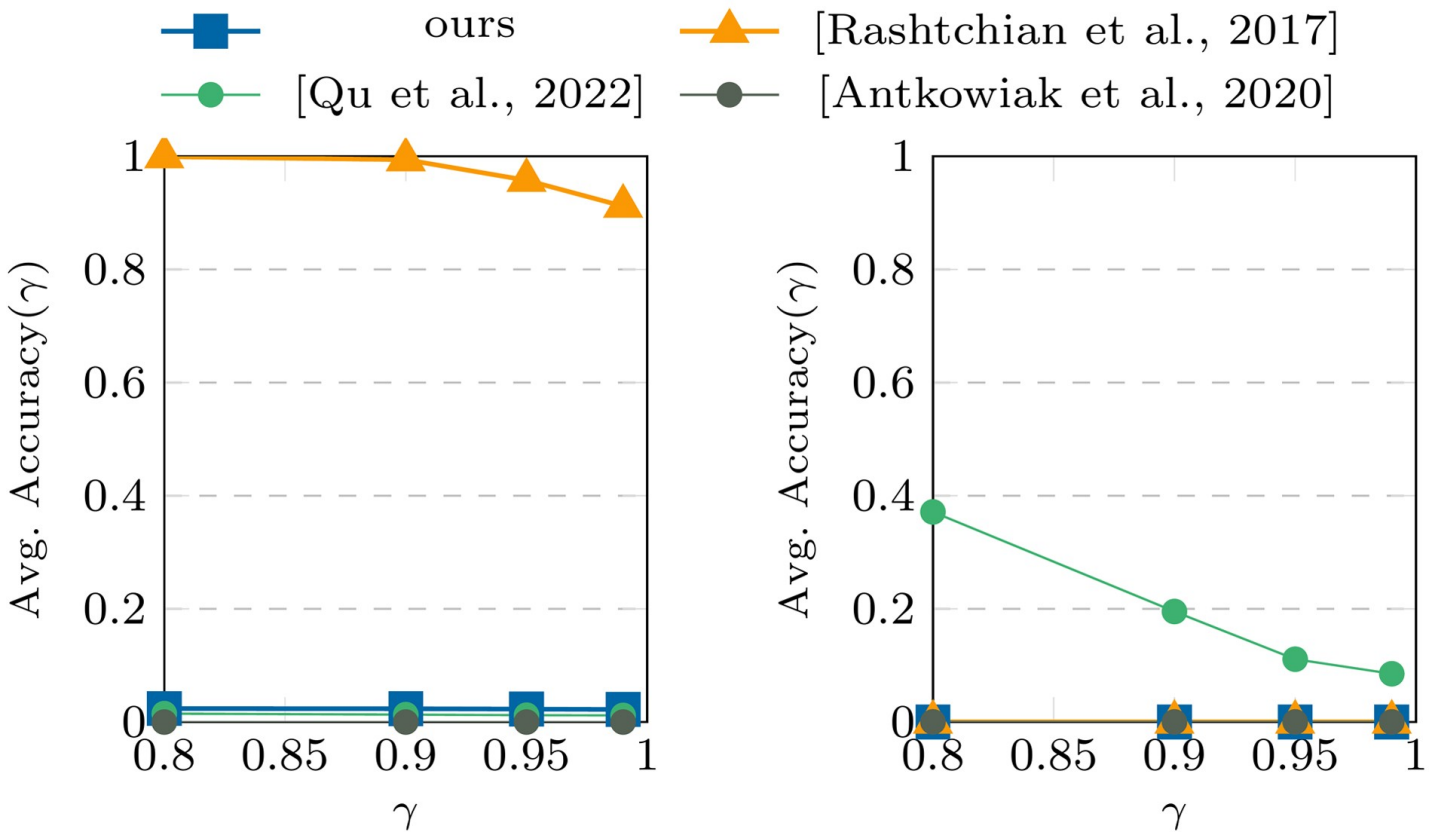
Accuracy (γ) results on datasets III and IV.

**Figure 5.**
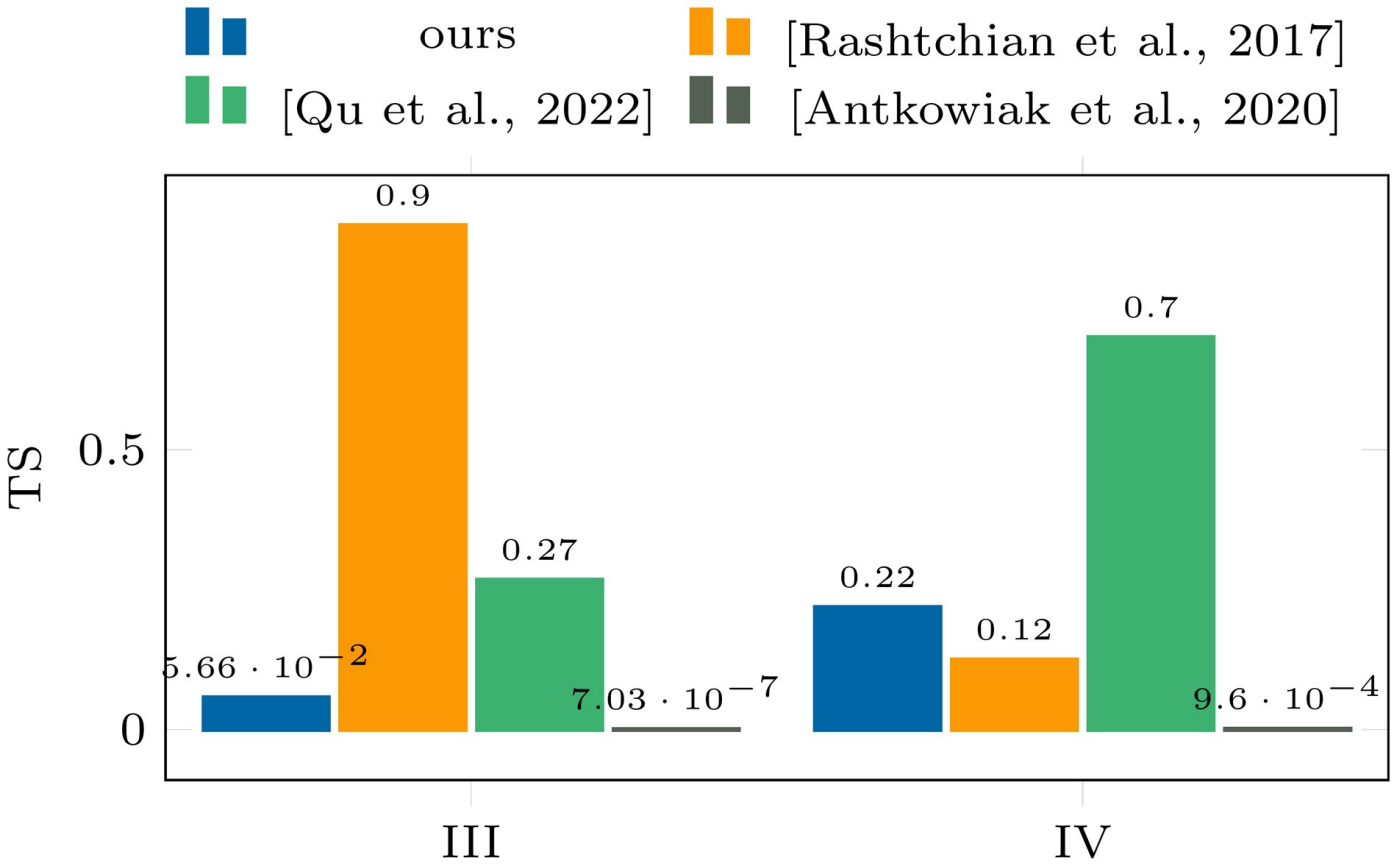
TS results on datasets III and IV.

Figures 4 and 5 show the performance results of the algorithms (for a single successful run) on datasets III and IV, respectively. Compared to the results from dataset I, the algorithms performed significantly worse. To address this issue, we conducted additional analysis of these datasets and proposed a solution based on pseudo-randomization.

#### 4.1.1 Enhancing clustering performance with pseudo-randomized designs

As mentioned before, all algorithms performed poorly on dataset IV. Moreover, with the exception of Rashtchian *et al.* (2017), all algorithms struggled to cluster dataset III. These results motivated us to conduct further analysis of these two datasets to identify what makes them difficult to cluster. We discovered that in the original design, many long runs of characters appear multiple times in different input strands. These patterns appeared in most of the noisy copies of the input strands. Such patterns have a significant impact on a crucial step in clustering algorithms: the assessment of similarity between two random input strands, particularly when it relies on examining the correlation of their substrings. This critical step enables algorithms to make informed decisions and assign two random input strands to the same original cluster. Therefore, we concluded that such designs are likely to lead clustering algorithms to complete failure.

Here, we propose a simple solution for such designs, requiring only a few extra bits of redundancy. Given an input design (with potential similarity among different DNA strands), one can randomly choose a seed and use it to generate pseudo-random DNA strands matching the original design’s length and input set size. Each input strand is then XORed with its corresponding pseudo-random DNA strand, ensuring a high likelihood that the new strands are far from each other (in terms of edit distance) and do not contain repeated substrings across different input strands. To retrieve the original data, pseudo-random strands are regenerated using the original seed and XORed with the received information. The scheme’s redundancy is log(seed)=O(1), as only extra bits are needed for the seed value.

**Figure 6.**
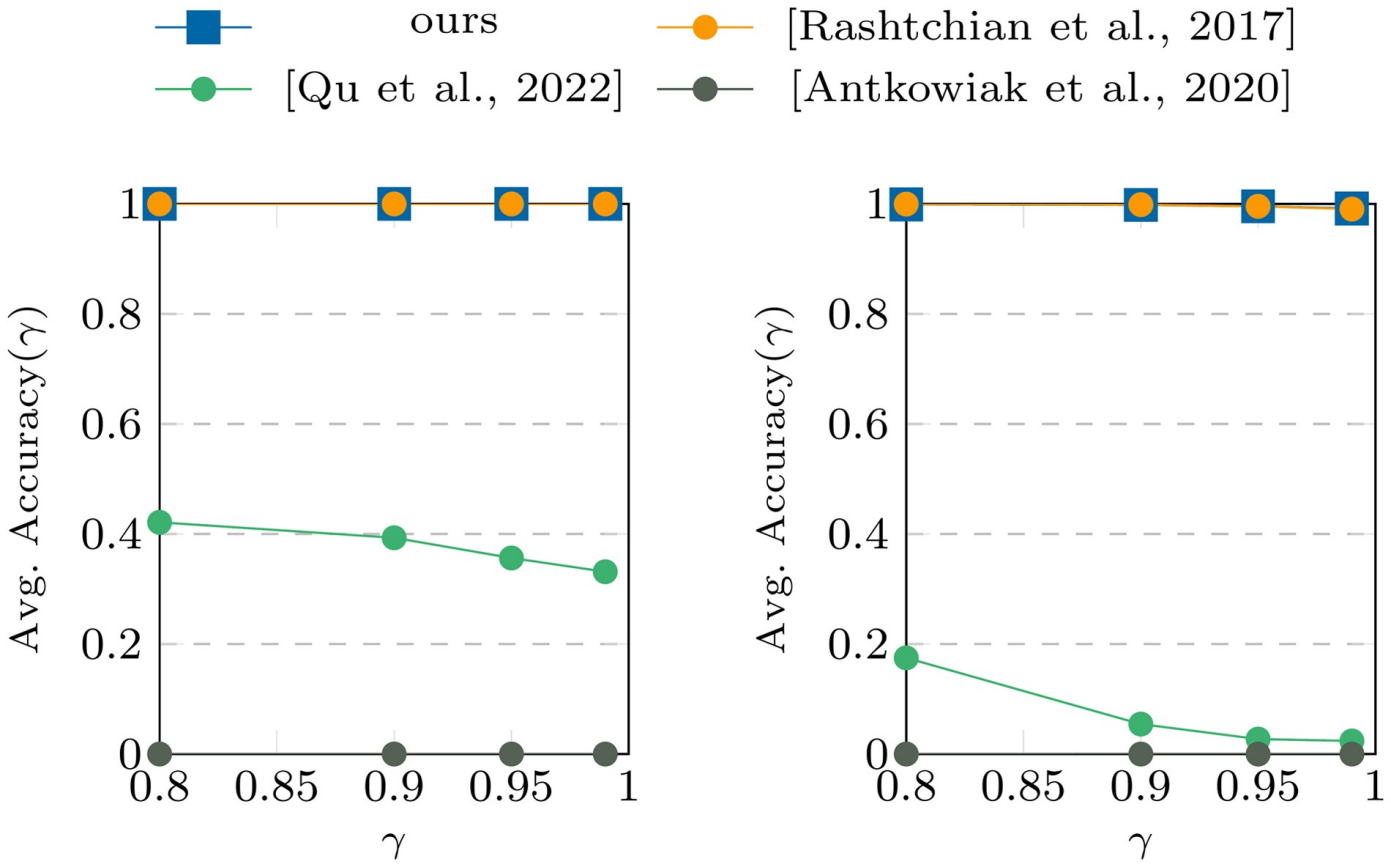
Accuracy (γ) results on datasets III^*^ and IV^*^. ^*^Perturbed datasets.

**Figure 7.**
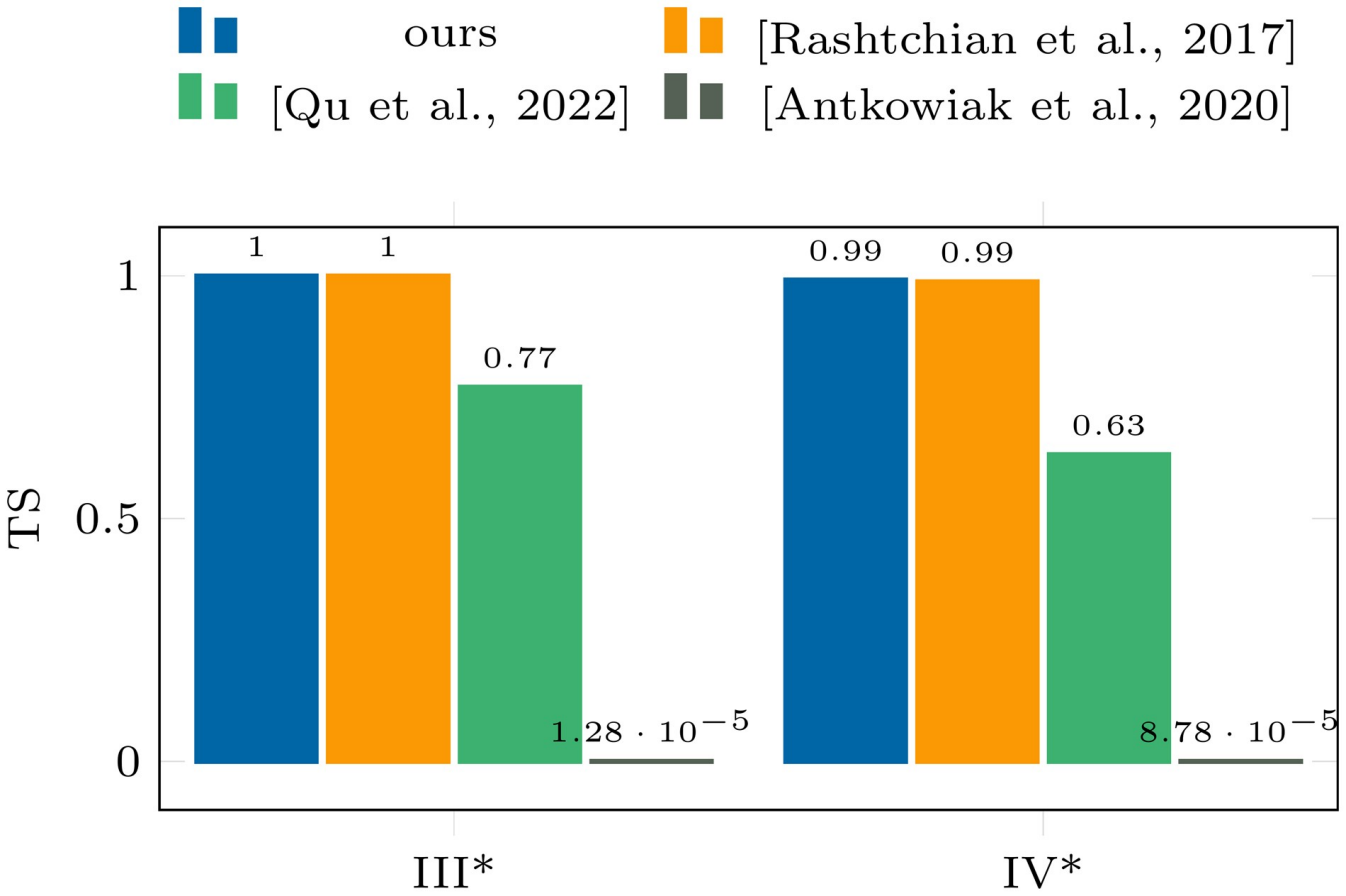
TS results on datasets III^*^ and IV^*^. ^*^Perturbed datasets.

To verify the correctness and efficiency of this technique, we applied it to the original designs of datasets III and IV. We XOR-wised the original designs with the pseudo-random strands and created clusters with noisy copies for each resulting strand. The cluster sizes were consistent with the original dataset’s cluster sizes. This process was performed using the DNA-Storalator (Chaykin *et al.* 2022) and was based on the error characterization of the original datasets. The results of this scheme are presented in Figs 6 and 7.

The comparison of the clustering algorithms in Figs 4 and 5 with those in Figs 6 and 7 shows that the proposed pseudo-randomization scheme is highly efficient and accurate, leading to a significant improvement in clustering performance. In addition, we applied the described technique to datasets I and II, where multiple long patterns were not present in the original design. As with datasets III and IV, the scheme resulted in improvements in the clustering results. Full details and results can be found in the Supplementary Material. Therefore, it is recommended to use this scheme, either alone or in combination with other encoding methods, for better results in the clustering phase.

### 4.2 Simulated datasets

All simulated datasets used were generated with uniform random data. The errors were simulated artificially, with values typical for the relevant synthesis technology. The error simulation process was performed using the DNA-Storalator (Chaykin *et al.* (2022), and based on error characterization analysis of the SOLQC tool (Sabary *et al.* 2020). Table 4 shows an extended summary of the simulated datasets used and their properties.

**Table 4.** Properties of simulated datasets.

	Data	Copies	Len.	Clusters	Err. (%)		
				Sizes	sub	ins	del
V	500 000	7 987 578	130	0–32	2.2	1.7	2
VI	500 000	8 002 591	60	0–32	2.2	1.7	2
VII	500 000	8 002 736	150	0–32	0.132	0.058	0.023

We randomly selected datasets V and VI to evaluate the performance of the algorithms through multiple runs (10).

**Figure 8.**
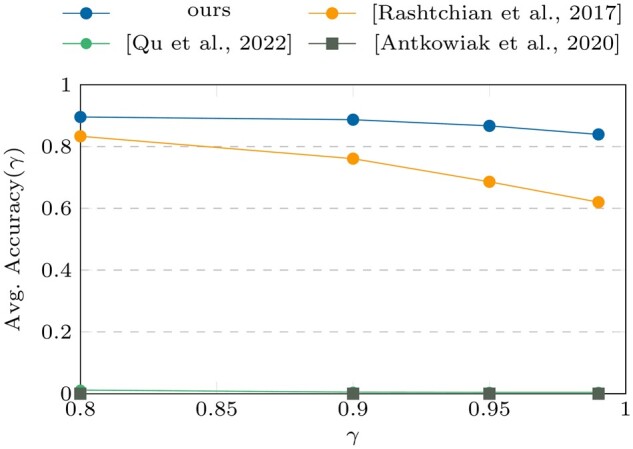
Avg. accuracy (γ) results on dataset VI.

**Figure 9.**
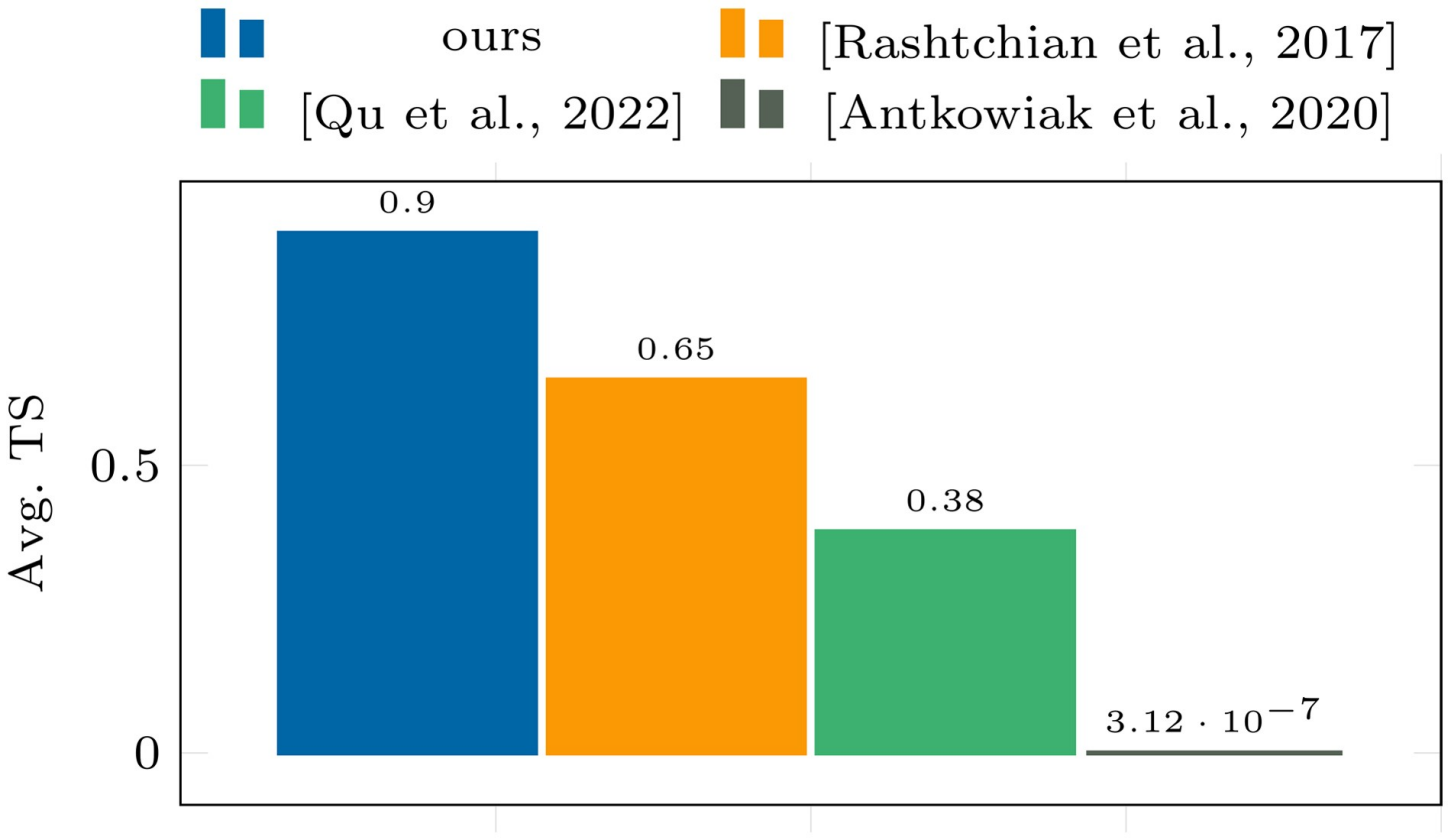
Avg. TS results on dataset VI.

As shown in Figs 8 and 9, our algorithm outperforms all other algorithms on dataset VI. Clover (Qu *et al.* 2022) and Antkowiak *et al.*’s clustering algorithm (Antkowiak *et al.* 2020) struggled to effectively cluster dataset VI, compared to both our algorithm and the one in Rashtchian *et al.* (2017). Tables 5 and 6 provide a summary of the performance metrics and running times of all the algorithms on this dataset. Comparing the results of all algorithms with those obtained from previous datasets, it is evident that all algorithms exhibited relatively poorer performance on dataset VI. As outlined in Table 4, this dataset was generated from very short input strands with a length of 60. Additionally, it comprises significantly smaller clusters in comparison to the cluster sizes in datasets I–IV. These two characteristics encounter challenges in the clustering process, due to a limited number of segments available for assessing similarity between the noisy copies. Furthermore, any misclassification becomes more significant due to the small cluster sizes.

**Table 5.** Results summary of dataset VI.

	Avg. acc.	SD acc.	Avg. TS	SD TS
	(γ=0.95)	(γ=0.95)		
Ours	**0.8669**	0.0059	**0.9036**	0.0042
Rashtchian *et al.* (2017)	0.6858	0.3176	0.6485	0.2808
Qu *et al.* (2022)	0.0042	8.66$\;\cdot \;10^{-5}$	0.3843	0.0002
Antkowiak *et al.* (2020)	0	0	3.12$\;\cdot \;10^{-7}$	0

**Table 6.** Runtime summary of dataset VI.

	Avg. runtime (*s*)	SD runtime (*s*)
Ours	9934	770.85
Rashtchian *et al.* (2017)	13525	9270.39
Qu *et al.* (2022)	**758**	**8.98**
Antkowiak *et al.* (2020)	3410	71.08

Full results of the algorithms on datasets V and VII can be found in the Supplementary Material.

### 4.3 Overall results summary

A comprehensive summary of the results for all algorithms on all datasets is found in the Supplementary Material.

## 5 Discussion

Our algorithm, along with the clustering algorithm in Rashtchian *et al.* (2017), produced the best results across all tested datasets. As expected, better clustering performance was achieved by all algorithms as the error rates decreased. In terms of runtime, Clover (Qu *et al.* 2022) outperformed all other algorithms by a significant margin. However, for DNA-based storage, designed for long-term archives (Grass *et al.* 2015, Ceze *et al.* 2019) (given the complexity and slowness of the synthesis and sequencing processes), clustering speed is not the top priority. In contrast, the LSH-based clustering algorithm (Antkowiak *et al.* 2020) performed poorly on all datasets, both simulated and experimental.

During benchmarking, the algorithm described in Rashtchian *et al.* (2017) exhibited unexpected behavior and instability, particularly with datasets containing small clusters. This instability is evident in high standard deviation values for clustering results and runtime (as shown in Tables 2, 3, 5, and 6).

In conclusion, the most significant advantage of our approach is to consistently deliver high-quality results, even with challenging inputs like short designs, small clusters, and high error rates. While the algorithm in Rashtchian *et al.* (2017) can sometimes produce similar clustering results to our algorithm in individual runs, its inconsistency poses a potential risk to the reliability of the DNA storage system.

More details on this behavior, key features of our proposal designed to overcome these issues, and further discussion on (Antkowiak *et al.* 2020, Qu *et al.* 2022), can be found in the Supplementary Material.

## Supplementary Material

btae274_Supplementary_Data

## Data Availability

GradHC is freely available at https://github.com/bensdvir/GradHC. Our implementation uses Python and includes both serial and parallel modes.
