# Supplementary material for: GradHC: highly reliable gradual hash-based clustering for DNA storage systems
Source: Bioinformatics. 2024 Apr 22;40(5):btae274. doi: 10.1093/bioinformatics/btae274 (PMC11653902; doi:10.1093/bioinformatics/btae274)
Supplement: btae274_Supplementary_Data [file btae274_supplementary_data.pdf]

GradHC: Highly Reliable Gradual Hash-based Clustering for DNA Storage Systems Supplementary Material

Dvir Ben Shabat, Adar Hadad, Avital Boruchovsky, and Eitan Yaakobi

Computer Science Department, Technion, Haifa, 3200003, Israel.

1 Related Work - Experiments and Methods

There are several DNA-clustering algorithms, where most of them are from the field of bioinformatics and metagenomics. UCLUST [14] and CD-HIT [15] are among the most used clustering methods, both of them use greedy algorithms for the clustering process while they rely on the Needleman-Wunsch global alignment algorithm for calculating the similarity between the sequences. DNACLUST [16] is another greedy clustering algorithm, that uses fast sequence alignment techniques and k-mer-based filtering. MeShClust v1.0 [17] uses the famous mean shift algorithm in order to overcome the challenge that when using a greedy algorithm there is no guarantee that an optimal solution can be found. MeShClust v3.0 [18] uses the mean shift algorithm and alignment-free identity scores for the sake of not paying precious time due to the costly global alignment algorithm. MetaDEC [19] applies a deep unsupervised learning approach to cluster metagenomic DNA strands. MMseqs2 [20] uses a graph-based approach, in which every noisy copy is a vertex, and two noisy copies are connected if they satisfy a particular similarity criteria.

One main weakness of some DNA-clustering algorithms, including those mentioned above, is that they rely on a single sequence identity threshold applied to every cluster. This biologically comprehensible parameter ranges from 0 (no sequence similarity) to 1 (identical strands). However, selecting an inappropriate identity threshold can lead to poor quality clusters. Determining the optimal threshold value requires users to have domain knowledge, which can be complex in DNA storage systems. ALFATClust [21] uses rapid pairwise alignment-free distance calculations and community detection to generate clusters, dynamically determining the cut-off threshold for each individual cluster based on cluster separation and intra-cluster sequence similarity. SEED [22] uses advanced hashing techniques and interval seeding to index DNA strands.

All of the algorithms mentioned above are designed for clustering genomic or meta-genomic data with the goal of grouping similar DNA strands into clusters, which can then be assembled. In DNA storage systems, the clustering step aims to divide the noisy copies so that each cluster can be independently reconstructed afterward. A key difference is that in DNA storage systems, the DNA strands are much shorter (50-200 nucleotides) compared to genomic data, which consists of long reads of tens of thousands of nucleotides. Therefore, these algorithms are not suitable for clustering short DNA strands.

Starcode [23] clusters the noisy copies based on an all-pairs search where it derives the Levenshtein distance[1] mainly by using the so-called edit matrices. This algorithm is designed to cluster DNA barcodes - very short DNA strands (tens of nucleotides at most). DBSCAN [24] is one of the earliest clustering algorithms, suggesting clustering together data samples based on their density and identifying outliers as noise. However, Guanjin et al. [25] have shown that this algorithm (as well as other density-based clustering algorithms) is impractical for clustering hundreds of thousands of noisy copies due to computational constraints. Shinkar et al. [26] proposed an index-based clustering algorithm and introduced a novel coding scheme called clustering-correcting codes, which uses both the data and index fields for the encoding process. Nonetheless, the success of this algorithm is guaranteed only under the strong assumption that the majority of strands in every generated cluster are derived from the same original strand. Another DNA clustering approach was presented in a recent work done in [27]. The authors presented a novel distributed algorithm that managed to cluster billions of reads in under one hour. The algorithm attractively merges clusters based on random representatives while using a hashing scheme to estimate the edit distance. Antkowiak et al. [28] demonstrated a DNA storage system that relies on massively parallel light-directed highly-error-prone synthesis. In their work, they presented an LSH-based

[1] Minimum number of single-character edits (insertions, deletions or substitutions) required to change one word into the other.

(locally sensitive hashing) clustering algorithm. This group managed to successfully store 100 KB of information. Clover is one of the newest DNA storage clustering algorithms available, developed by Guanjin et al. [25]. This algorithm has a linear computational complexity and low memory consumption. It achieves its speed by avoiding computation of the Levenshtein distance and using a tree structure for interval-specific retrieval instead.

2 Performance Validation

2.1 Review of Other Commonly Used Evaluation Metrics

Another clustering evaluation metric was presented in [25]. The authors defined the accuracy of two clusterings C and $\widetilde{C}$ by:

$$Accuracy(C, \widetilde{C}) = \frac{1}{|C|} \sum_{k} \max_{j} |\widetilde{C}_k \cup C_j|$$

where $C = (C_1, C_2, \ldots, C_j)$ refers to the perfect clustering and $\widetilde{C} = (\widetilde{C}_1, \widetilde{C}_2, \ldots, \widetilde{C}_k)$ is the output of their clustering algorithm. One can notice that a naive clustering algorithm, which clusters every noisy copy in its own cluster, gets a maximum score for the above metric. For this reason, we chose not to use this metric as part of our performance validation.

Other common metrics for clustering algorithm evaluation are *Purity* and Normalized-Matual-Information (*NMI*) [21]. *Purity* does not penalize misclassifications within clusters and favors clustering solutions with a larger number of clusters, even if they are not meaningful. Therefore, we have chosen not to use this metric in the evaluation process. In several cases, *NMI* may be influenced more by the sizes of the clusters than by the actual quality of the clustering. This is because *NMI* assumes balanced data point distribution (which implies cluster sizes are roughly the same), making it unsuitable for DNA storage systems with unevenly distributed cluster sizes.

Here are two examples illustrating issues when using the NMI metric. Let C denote a clustering result represented as a vector of indices, where $C[i]$ represents the index of the cluster to which S_i belongs in the clustering:

Example 1: Assume the true clustering is $C_{\text{original}} = [1, 1, 1, 2, 2, 2, 3, 3, 3, 4, 4, 4]$, and that the output clustering, which lacks an entire cluster and has several misclassifications, is $C_{\text{output}} = [1, 1, 1, 1, 1, 1, 2, 2, 2, 3, 3, 3]$. The *NMI* of these two clusterings is $NMI(C_{\text{original}}, C_{\text{output}}) = 0.8571$, indicating a relatively good clustering results. However, reconstructing the first cluster may pose challenges due to the frequent occurrence of false-positives, and the last input strand remains unrecoverable in the clustering output. This illustrates that, although *NMI* may suggest relatively good clustering results, it might not necessarily reflect the true quality of the output. In this instance, both Accuracy(γ) (for any value of γ) and TS will produce results closely resembling the quality of the output clustering, with values of 0.5 and 0.75, respectively.

Example 2: Assume the true clustering is $C_{\text{original}} = [1, 1, 1, 1, 1, 2, 2, 2, 2, 2, 3, 3, 3]$, and the output clustering, containing a single strand misclassification in two major clusters, is $C_{\text{output1}} = [\mathbf{2}, 1, 1, 1, 1, 2, 2, 2, 2, 2, 3, 3, 3]$. The *NMI* of these two clusterings is $NMI(C_{\text{original}}, C_{\text{output1}}) = 0.812$. On the other hand, consider another output clustering, which contains a single strand misclassification but in a much smaller cluster: $C_{\text{output2}} = [1, 1, 1, 1, 1, 2, 2, 2, 2, 2, 3, 3, \mathbf{1}]$. The *NMI* of these two clusterings is $NMI(C_{\text{original}}, C_{\text{output2}}) = 0.829$, which is greater compared to $NMI(C_{\text{original}}, C_{\text{output1}})$. However, it is important to note that the misclassification in this example is much more significant since reconstructing the last input strand becomes much harder.

From the examples mentioned above, we have chosen not to include *NMI* as part of the performance validation process.

2.2 Accuracy(γ) and TS as the Most Optimal Clustering Evaluation Metrics for DNA Storage Systems

The *Accuracy(γ)* metric allows us to get a decisive indication of the percentage of clusters that managed to restore so that a good *reconstruction algorithm* will succeed in reconstructing the original strand from them. This is due to the fact that they contain most of the original cluster, and do not contain noisy copies of another input strand. According to the analysis in [13], reconstruction algorithms yield significantly poor results for clusters that are either too small or too noisy. Therefore, it can be inferred that low *Accuracy(γ)* values of a clustering algorithm directly lead to inaccurate/unfeasible reconstruction of certain clusters in the dataset, negatively impacting the reconstructability of the original data.

The *TS* metric allows us to get a high-level perspective on the full clustering result. In general, a high *TS* value implies two important conclusions:

1. The number of clusters recovered is high - considering the relatively small number of false-negatives.
2. The generated clusters were recovered with high quality since the number of false-positives is low compared to the number of true-positives.

From all of the above, we chose to use Accuracy(γ) and TS for evaluating the clustering results, as we find them to be the most suitable and precise for the validation of clustering algorithms for DNA storage systems.

3 The GradHC Algorithm

3.1 Parameter Selection

Step 1 - Division into Chunks: We set the values for w and t to be $w = \lceil \log_4 l \rceil$, and $t = \log_4 n - w$, as recommended in [27]. The values for *mergers_ratio* and p (number of ineffective rounds) were chosen empirically and set to 0.0002 and 3, respectively.

Step 2 - Clustering per Chunk: We have control over three parameters - q (the size of the q-gram), k (the length of the *LSH signature*), and the number of iterations of the algorithm. Antkowiak et al. [28] suggest that setting the signature's length around 3 helps to reflect enough information from the original strand, while still allowing for justified merging decisions later. From similar arguments, a suitable range of values for q would be between 3 and 7. As stated above, the probability of two strands s_i and s_j to have the same *MinHash* signatures relates to the desired similarity of their number sets. The values used as thresholds for determining similarity are $\theta_{high} = 0.32$ and $\theta_{low} = 0.28$. The motivation behind the usage of those values is an attempt to find a balance between wanting the sequences to be close enough (resulting in similar number sets, and a high Sørensen–Dice similarity), without missing too many related sequences for not being perfectly identical. Following the above, it can be concluded that the probability of two strands with similar number sets having the same signatures is $similarity^k$. When using $k = 3$ such signatures, and the high similarity threshold of $\theta_{high} = 0.32$, the number of *LSH signatures* that the algorithm needs to compute to ensure finding all pairs of similar strands s_i and s_j with high probability is denoted by: $\left(\frac{1}{similarity}\right)^k = 1/0.32^3 \approx 30$. Therefore, we set the required number of iterations to 30.

Step 3 - Full Clustering: In this step, the algorithm uses two values, namely $\theta_{high} = 0.25$ and $\theta_{low} = 0.22$, as thresholds to determine the similarity between clusters. Lower thresholds are used as the algorithm now attempts to handle strands that could not be clustered in the previous step, implying they are likely to be more erroneous.

3.2 Time Complexity of the Algorithm and Implementation Notes

In this section, we show that the run time for our proposed algorithm is $O(nl)$ in the worst case, where n is the number of sequences in the input, and l is the strand's length. In the running time analysis of the algorithm, we pay attention to each step on its own.

When splitting the input into chunks in Step 1, the number of iterations is dynamically determined during the run. However, we also use a constant upper bound, not allowing it to exceed a certain number of iterations. The value of this upper bound was deduced empirically. In each iteration, we compare the clusters' signatures. Both computing the signatures and executing the comparisons are performed in linear time ($O(l)$). In practice, using representatives from chunks instead of iterating over all the sequences contributes to a significant performance improvement.

Creating a number set and an *LSH signature* for a single strand depends on its length. Therefore, the setup essential for the entire clustering process in Step 2 is $O(nl)$. During the clustering process, the sequences are compared using the signatures, but unlike Step 1, an additional check of the similarity is added. Nevertheless, it demands at most $O(l)$ operations.

The last step is technically identical, aside from refraining from going through the whole input. Instead, we use $O(1)$ representatives from each cluster. For this reason, the upper bound remains the same, although the actual run time dramatically improves.

According to the design of the algorithm, several steps can take advantage of multi-core processors. For example, the computation of the number sets and the *LSH signatures* does not require any kind of communication between the cores and can benefit from parallelism. Additionally, in Step 2, the clustering is done in each chunk on its own, suggesting that it is also possible to perform this step in parallel. In our implementation, we support parallel mode which parallelizes some of the steps mentioned above.

3.3 Local-Sensitive-Hashing and Sørensen-Dice Coefficient as Proxies for Normalized-Edit-Distance(NED)

To assess the similarity between two sequences s_i, s_j, and determine whether they belong to the same cluster in Steps 2 and 3 of the algorithm, we utilize the LSH signatures of the sequences. Additionally, we approximate the similarity of their numsets (N_{s_i}, N_{s_j}) using the Sørensen–Dice coefficient [33]. In this section, we dive into the correlation between these metrics for two sequences and their corresponding Normalized-Edit-Distance (NED) values. The algorithm requires the *LSH signatures* (of length k) of two sequences to be equal before setting them in the same cluster. The probability of two sequences s_i, s_j to have the same *LSH signatures* is defined by:

$$Pr[LSH_{s_i} = LSH_{s_j}] = (Pr[sigs[s_i] = sigs[s_j]])^k \text{ , where } Pr[sigs[s_i] = sigs[s_j]] = \frac{\sum_{r=1}^{|sigs[s_i]|} 1\{sigs[s_i][r] = sigs[s_j][r]\}}{|sigs[s_i]|}.$$

Using the DNA-Storalator [35], we generated two sample datasets, each containing 500 and 700 noisy copies overall, respectively. The error rates employed while generating the datasets are the same as in dataset V. For every pair of sequences s_i, s_j in the dataset, we derive three values: $\texttt{NED}(s_i, s_j)$, $\texttt{sørensen\_dice}(N_{s_i},\ N_{s_j})$, and their probability to have the same *LSH signatures*, $Pr[LSH_{s_i} = LSH_{s_j}]$.

Figures 1 and 2 illustrate the relationship between the probability of having the same *LSH signatures* and NED values, while Figures 3 and 4 show the correlation between Sørensen-Dice values and NED values.

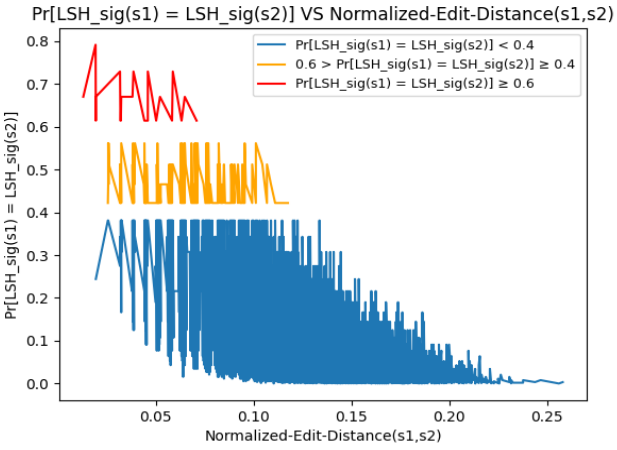

Figure 1: Correlation between LSH to NED on sample dataset 1.

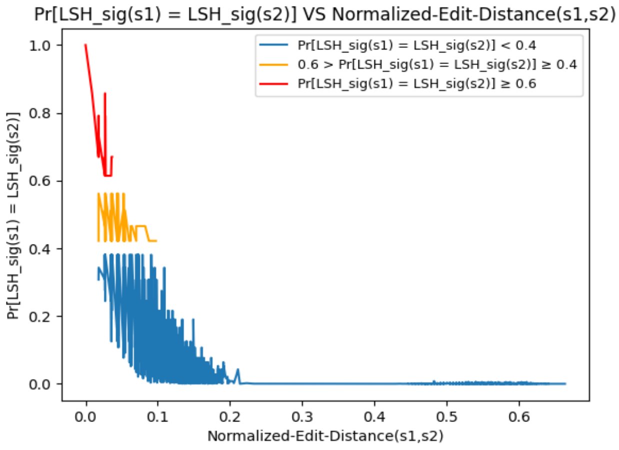

Figure 2: Correlation between LSH to NED on sample dataset 2.

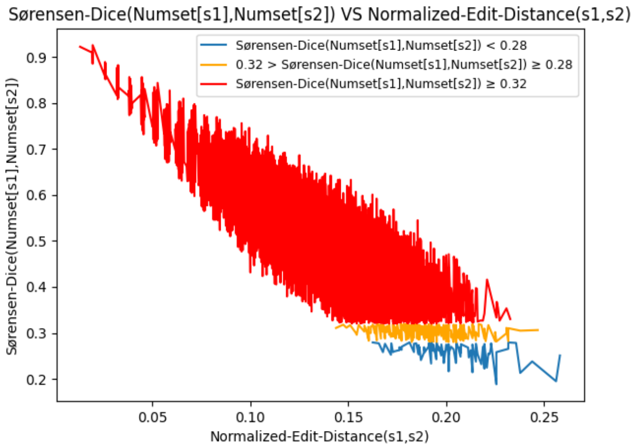

Figure 3: Correlation between Sørensen-Dice to NED on sample dataset 1.

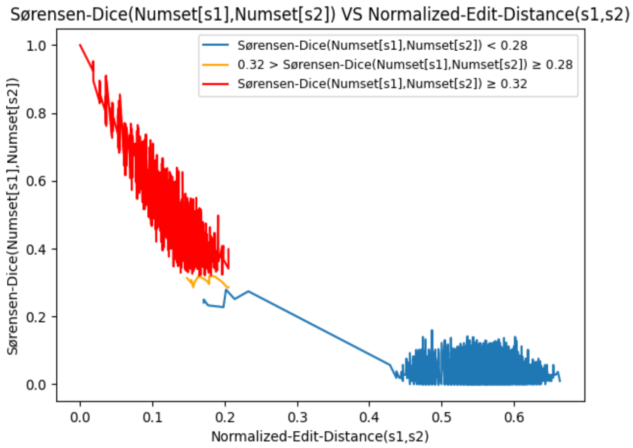

Figure 4: Correlation between Sørensen-Dice to NED on sample dataset 2.

After generating chunks in Step 1, we use the comparison of *LSH signatures* as a key condition, although not comprehensive, for assigning two sequences to the same cluster. Figures 1 and 2 highlight a significant observation: for all pairs of sequences s_i and s_j, where $Pr[LSH_{s_i} = LSH_{s_j}] \geq 0.8$, it holds that $\texttt{NED}(s_i, s_j) \leq 0.1$. Moreover, we assert that sequences having the same random *LSH signature* in a given iteration of the algorithm are highly likely to display elevated values of $Pr[LSH_{s_i} = LSH_{s_j}]$. Consequently, they are highly probable to belong to the same cluster. Furthermore, it can be inferred that sequences s_i and s_j that do not share the same *LSH signature* in a given iteration (and thus it is likely that $Pr[LSH_{s_i} = LSH_{s_j}]$ is low) exhibit a relatively large normalized edit distance, suggesting a high probability that they originated from different input strands (and have been filtered out correctly by our algorithm).

It is important to note that comparing a single *LSH signature* of two sequences alone is not sufficient in practice. Since we refer to a single *LSH signature* in each iteration of Steps 2 and 3, collisions may occur. While the connection between the probability of having the same *LSH signatures* and NED values is a motivating factor, it conceals the potential for false-positives. In scenarios of low probability (though they do exist), we might come across a pair of sequences s_i and s_j with a low probability of having $Pr[LSH_{s_i} = LSH_{s_j}]$. In such cases, and based on Figures 1 and 2, the normalized edit distance of the two sequences, $\texttt{NED}(s_i, s_j)$, will be much higher. Therefore, we introduce a second condition before merging the clusters of those sequences.

In Step 2 of the algorithm, we applied similarity thresholds, using $\theta_{high} = 0.32$ and $\theta_{low} = 0.28$. In Step 3, we adopted softer thresholds: $\theta_{high} = 0.25$ and $\theta_{low} = 0.22$. Figures 3 and 4 demonstrate that any pair of sequences s_i, s_j with a Sørensen-Dice coefficient of their numsets greater than 0.25 ensures a NED of 0.2 or less. Thus, a strong correlation exists between the Sørensen-Dice coefficient of the numsets for two sequences s_i, s_j and the corresponding NED value. By selecting sufficiently tight thresholds on the similarity between numsets of two sequences, we can estimate with a high probability that the normalized edit distance between them is small. This implies a strong likelihood that the sequences originated from the same input strand.

4 Additional Results

4.1 Experimental Datasets

4.1.1 Experiments Details and Background

Our experimental datasets were collected from real DNA storage experiments performed in recent years:

I. 2015, Grass et al. [3] - This group encapsulated DNA in an inorganic matrix, and employed error-correcting codes to correct storage-related errors. Specifically, they translated 83KB of information to 4991 DNA strands, each 158 nucleotides long (117 nucleotides of encoded information and 41 nucleotides served as adapters), which were encapsulated in silica.

II. 2017, Erlich and Zielinski [8] - In this work, the researchers encoded 2.11MB of data into 72,000 DNA sequences of length 152. The coding scheme, "DNA Fountain", was based on the Luby transform with redundancy of 7% (means that 67,088 sequences were information sequences, and the rest were redundancy). The decoding is done with a message-passing decoder ("LDPC"-like). They also applied homopolymer and GC-content constraints in their code.

III. 2018, Organick et al. [4] - They encoded 200MB of data and published one file of 9.5MB of data which was encoded to 607,150 DNA sequences. In their coding scheme, they used RS code with 15% redundancy on the symbols. They also used primers to enable random access.

IV. 2021, Yekhanin et al. [9] - The paper introduces a new reconstruction algorithm - Trellis BMA, whose complexity is linear in the number of traces. For their performance comparisons, they published a new dataset of 269,709 traces of 10,000 uniform random DNA sequences of length 110 each.

Figures 5 and 6 present the full results of the algorithms on dataset II for a single successful run. Similarly to dataset I, it can be seen that our algorithm outperforms all other algorithms. The running times of the algorithms on this dataset are detailed in Table 5, which is presented in Section 4.3.

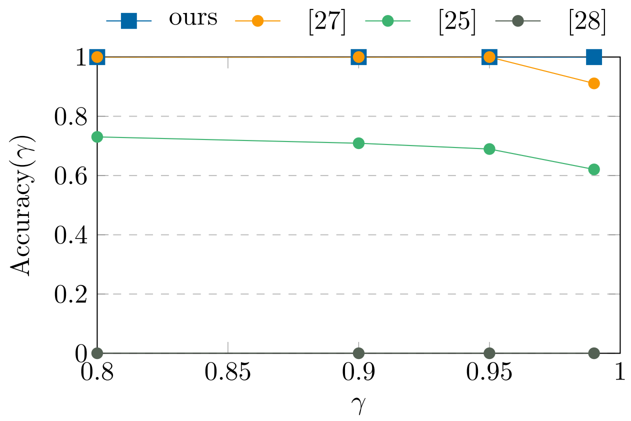

Figure 5: Accuracy(γ) results on dataset II.

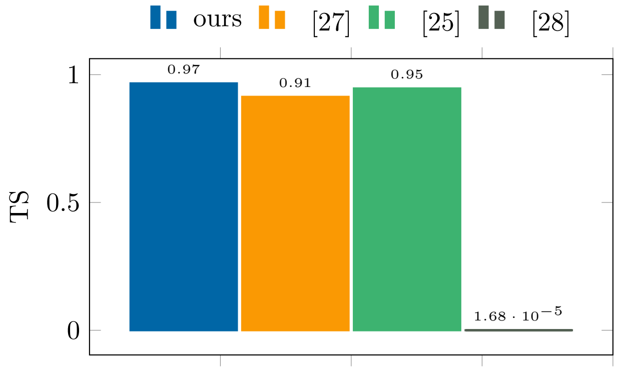

Figure 6: TS results on dataset II.

4.1.2 Enhancing Clustering Performance with Pseudo-Randomized Designs

As mentioned earlier, all algorithms performed poorly on dataset IV. Furthermore, except for [27], all algorithms struggled to cluster dataset III. To address this issue, we proposed a pseudo-randomized-based scheme, which significantly improved the clustering results for all algorithms on these datasets. Additionally, we applied the described technique to datasets I and II, where multiple long patterns were not present in the original design. This was done to verify the utility and correctness of the suggested scheme for all types of designs. As evidenced in Figures 7-10 and Tables 1-2, the scheme led to improvements in the clustering results for both datasets I and II. The TS values of the algorithms and their running times on the perturbed datasets can be seen in Section 4.3.

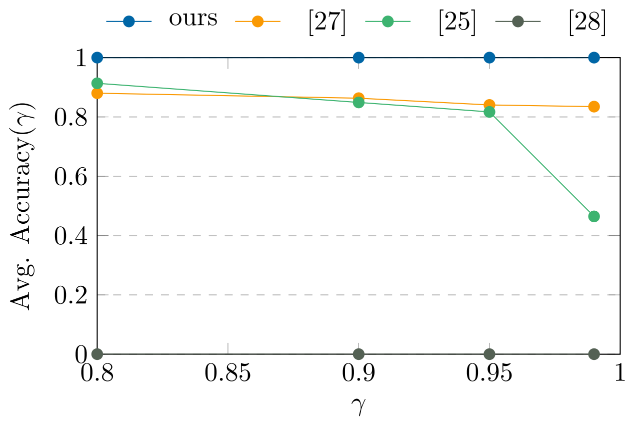

Figure 7: Avg. Accuracy(γ) results on dataset I*.
* perturbed dataset.

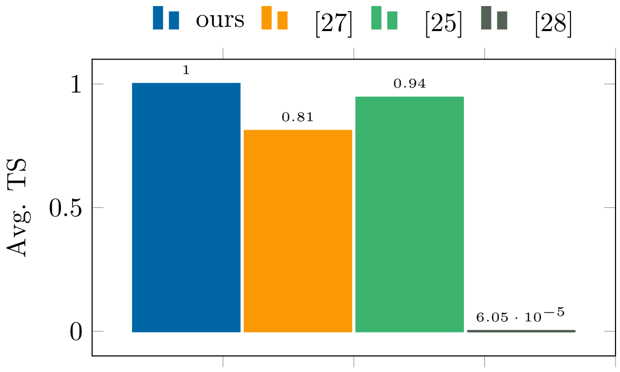

Figure 8: Avg. TS results on dataset I*.
* perturbed dataset.

	Avg. Acc. ($\gamma = 0.95$)	Stdev. Acc. ($\gamma = 0.95$)	Avg. TS	Stdev. TS
ours	**1**	0	**1**	0
[27]	0.8403	0.3908	0.8099	0.3956
[25]	0.8169	0.0058	0.9445	0.0018
[28]	$1.95 \cdot 10^{-4}$	$2.51 \cdot 10^{-4}$	$6.05 \cdot 10^{-5}$	$4.87 \cdot 10^{-5}$

Table 1: Results summary of dataset I*.
* perturbed dataset.

	Avg. Runtime (s)	Stdev. Runtime (s)
ours	2287	84.66
[27]	4175	4857.76
[25]	**48**	**1.87**
[28]	1225	7.13

Table 2: Runtime summary of dataset I*.
* perturbed dataset.

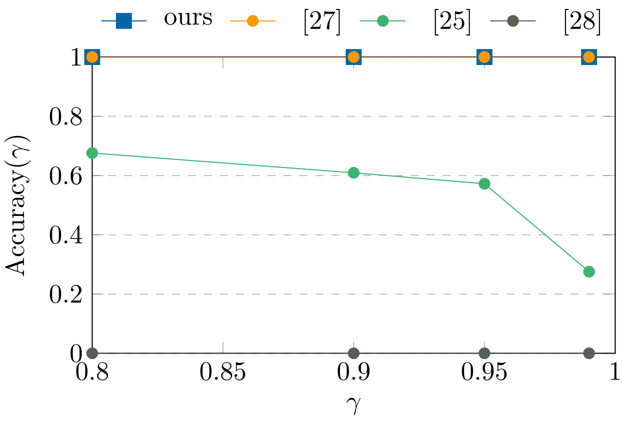

Figure 9: Accuracy(γ) results on dataset II*.
* perturbed dataset.

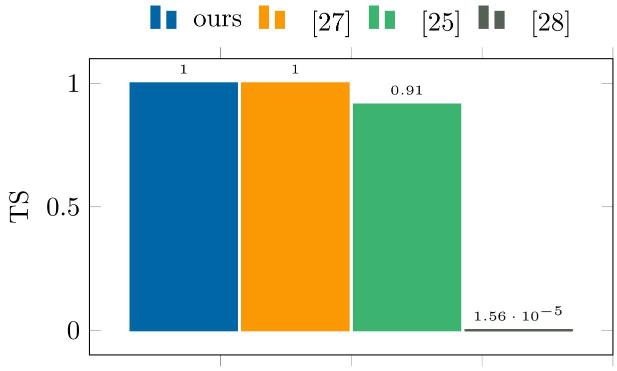

Figure 10: TS results on dataset II*.
* perturbed dataset.

4.2 Simulated Datasets

The evolution of the algorithms on dataset V refers to the results of 10 runs of each algorithm on the dataset. In Figures 11 and 12, it can be clearly seen that our algorithm outperforms all other algorithms. The summary of the average results and the values of the standard deviations between all the runs appear in Tables 3 and 4.

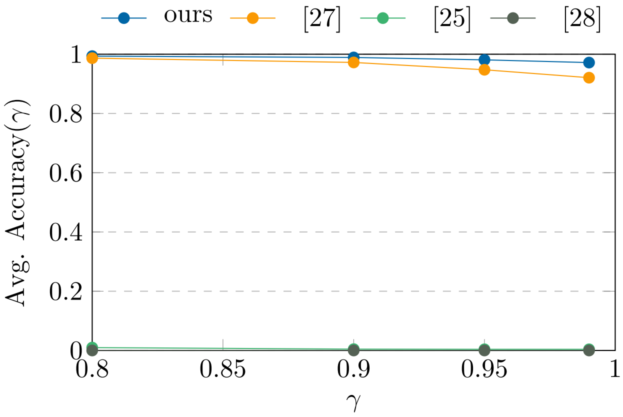

Figure 11: Avg. Accuracy(γ) results on dataset V.

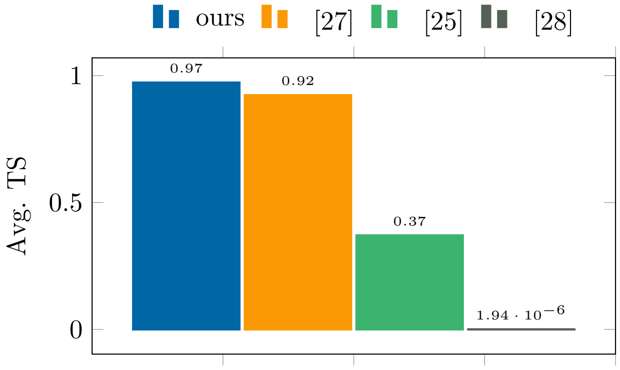

Figure 12: Avg. TS results on dataset V.

	Avg. Acc. ($\gamma = 0.95$)	Stdev. Acc. ($\gamma = 0.95$)	Avg. TS	Stdev. TS
ours	**0.9809**	0.0017	**0.9726**	0.0022
[27]	0.9475	0.0919	0.9229	0.1279
[25]	0.0039	$9.45 \cdot 10^{-05}$	0.3707	0.0003
[28]	0	0	$1.95 \cdot 10^{-6}$	0

Table 3: Results summary of dataset V.

	Avg. Runtime (s)	Stdev. Runtime (s)
ours	12946	452.12
[27]	18783	14242.22
[25]	**776**	**9.58**
[28]	3513	57.81

Table 4: Runtime summary of dataset V.

Figures 13 and 14 present the full results of the algorithms on dataset VII for a single successful run. As can be seen, our algorithm and the clustering algorithm in [27] produced the best clustering results on this dataset. The running times of the algorithms on this dataset are detailed in Table 5, which is presented in Section 4.3.

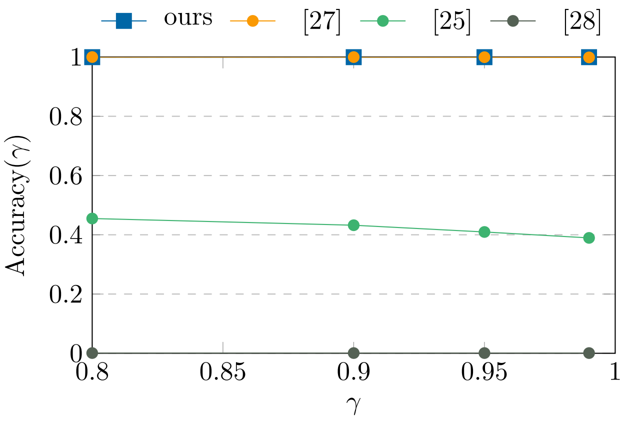

Figure 13: Accuracy(γ) results on dataset VII.

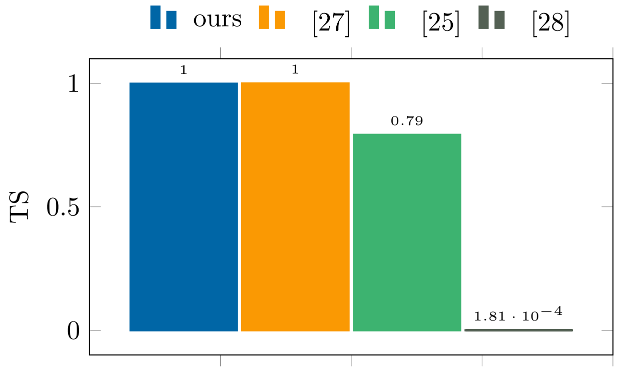

Figure 14: TS results on dataset VII.

4.3 Overall Results Summary

Figure 15 summarizes a comprehensive overview of the TS results for all datasets, highlighting trends and enabling clear comparisons between the general correctness of all the algorithms. Table 7 summarizes the running times of the algorithms.

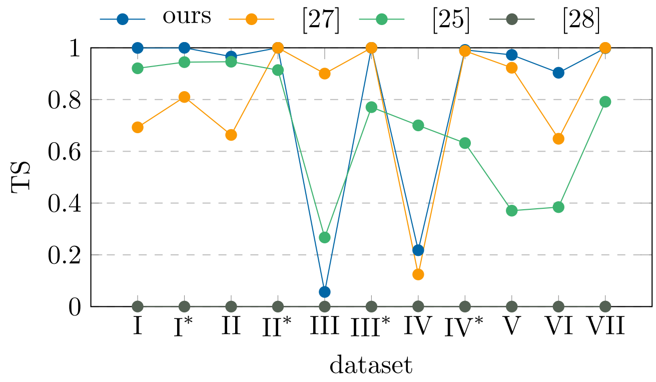

Figure 15: TS results on all datasets.* perturbed datasets

dataset	ours	[27]	[25]	[28]
I	2440**	7538**	**47****	1177**
I*	2287**	4175**	**48****	1225**
II	11692***	13280	**265**	6119
II*	7657***	11733	**284**	5989
III	9074***	11856	**1246**	4796
III*	8801***	9989	**671**	5111
IV	168	208	**18**	102
IV*	155	317	**18**	104
V	12946**	18783**	**776****	3513**
VI	9934**	13525**	**758****	3410**
VII	11637	14307	**554**	3444

Table 5: Runtime summary of all datasets (s).
* perturbed datasets, ** average running times,
*** runs over 128-cores AMD EPYC 7513 CPU, 1TB RAM.

5 Extended Discussion

5.1 Results Analysis of Clover [25] and the Clustering Algorithm by [28]

The algorithms that produced the best results on all tested datasets are ours and the clustering algorithm in [27]. As expected, better clustering performance was achieved by all algorithms as the error rates decreased. In terms of runtime, Clover [25] emerged as the fastest clustering algorithm, outperforming all other algorithms by several orders of magnitude. Clover achieves its speed by constructing a tree structure to search for a specified interval, instead of computing the Levenshtein distance between strands. However, despite its impressive speed, Clover was only able to achieve high-quality clustering results on datasets I and II, which are characterized by relatively low error rates and very large clusters. Furthermore, due to the complexity and slowness of the synthesis and sequencing processes involved in DNA-based storage, it is mainly intended for long-term storage such as archives [2, 3]. Thus, clustering speed is not the highest priority in such systems. In contrast, the LSH-based clustering algorithm [28] showed poor performance on all tested datasets, both simulated and experimental. It is important to note that although the algorithm's results were unsatisfactory, Antkowiak et al. [28] were able to recover all the information encoded in the experiment by applying coarse filtering on the clusters created and by using an outer code that could handle a high percentage of missing strands.

5.2 Key Features to Ensure Stability and Addressing the Algorithm Performance of [27]

During the benchmark performance, we observed that the implementation of [27] often exhibited unexpected behavior, whereas our algorithm and all other suggestions remained stable. To demonstrate the weakness of the algorithm in [27], we conducted multiple runs of the algorithm on datasets I, V, and VI (selected arbitrarily). This observation is reflected in the algorithm's TS and Accuracy(γ) standard deviation values, as shown in Tables 1 and 3 and Tables 2 and 5 in the paper. As can be seen, the standard deviation values of the clustering algorithm in [27] are several orders of magnitude greater than the standard deviation values of the other algorithms, which are close to 0. Additionally, when the algorithm fails to converge, its instability can significantly affect its running time. This is evident in the high standard deviation values of the algorithm's runtime (see Tables 2 and 4 and Tables 3 and 6 in the paper).

Occasionally, the clustering algorithm suggested in [27] failed to converge, impacting both the runtime and the metrics used to evaluate the measurements. Our hypothesis is that datasets containing small clusters may cause a situation where a series of incorrect merges in the early stages is sufficient for hurting the rest of the process. The reason originates from the fact that not all the strands participate in every iteration, and only representatives (whose selection is random) are used, making the algorithm vulnerable if poor decisions were made at the start. A key feature of our proposal appears in Step 2, in which the clustering process is done with the majority of the sequences taking part, instead of picking representatives. In addition, when representatives are used in Step 3, they are selected intelligently, according to their score. In this manner, we were able to overcome scenarios that could have failed the algorithm presented in [27].

In conclusion, the most significant advantage of our proposed approach is its ability to deliver consistent high-quality results, even when processing challenging inputs, such as short designs, small clusters, and high error rates. Although the algorithm in [27] can produce results (in terms of both runtime and correctness) that are comparable to or better than those of our algorithm in multiple runs, it often fails to provide satisfactory outcomes, as evidenced by the standard deviation values obtained from multiple runs of the algorithm.

References

[1] N. Goldman et al. "Towards practical, high-capacity, low-maintenance information storage in synthesized DNA", *Nature*, vol. 494, pp. 2013.

[2] L. Ceze, J. Nivala, K. Strauss, "Molecular digital data storage using DNA", *Nature Reviews Genetics*, vol. 20, pp. 456–466, 2019.

[3] R.N. Grass et al. "Robust chemical preservation of digital information on DNA in silica with error-correcting codes", *Angewandte Chemie International Edition*, vol. 54, pp. 2552–2555, 2015.

[4] L. Organick et al. "Random access in large-scale DNA data storage", *Nature Biotechnology*, vol. 36, pp. 242, 2018.

[5] G. M. Church , Y, Gao, S. Kosuri, "Next-generation digital information storage in DNA", *Science*, vol. 337, pp. 1628-1628, 2012

[6] S. Kosuri, G. Church, "Large-scale de novo DNA synthesis: technologies and applications", *Nature Methods* vol. 11, pp. 499–507, 2014

[7] S. Crosby et al. "Oligonucleotide and nucleic acid synthesis", *Patent*, 2019.

[8] Y. Erlich and D. Zielinski, "DNA fountain enables a robust and efficient storage architecture", *Science*, vol. 355, pp. 950–954, 2017.

[9] S. R. Srinivasavaradhan et al. "Trellis BMA: Coded trace reconstruction on IDS channels for DNA storage", *ISIT*, pp. 2453-2458, 2021.

[10] T. Batu et al. "Reconstructing strings from random traces", *SIAM*, pp. 910–918, 2004.

[11] P. S. Gopalan et al. "Trace reconstruction from noisy polynucleotide sequencer reads", *US Patent App*, 2018.

[12] K. Viswanathan and R. Swaminathan, "Improved string reconstruction over insertion-deletion channels," *SIAM*, pp. 399–408, 2008

[13] O. Sabary et al. "Reconstruction algorithms for DNA-storage systems," *bioRxiv*, 2020.

[14] R.C. Edgar, "Search and clustering orders of magnitude faster than BLAST", *Bioinformatics*, vol. 26, pp. 2460–2461, 2010.

[15] L. Fu et al. "CD-HIT: accelerated for clustering the next-generation sequencing data", *Bioinformatics*, vol. 28, pp. 3150–3152, 2012

[16] M. Ghodsi, B. Liu, M. Pop, "DNACLUST: accurate and efficient clustering of phylogenetic marker genes" *BMC Bioinformatics* vol. 12, pp. 271, 2011

[17] B. T James et al. "MeShClust: an intelligent tool for clustering DNA sequences", *Nucleic Acids Research*, Vol. 46, 2018

[18] H. Z. Girgis, "MeShClust v3.0: High-quality clustering of DNA sequences using the mean shift algorithm and alignment-free identity scores", *BMC Genomics*, vol. 23, 2022

[19] H. Q. Bao, L. V. Vinh and T. Van Hoai, "A Deep Embedded Clustering Algorithm for the Binning of Metagenomic Sequences", *IEEE Access* vol. 10, pp. 54348-54357, 2022

[20] M. Steinegger, J. Söding, "Clustering huge protein sequence sets in linear time", *Nature Communications* vol .9, pp. 2542 ,2018

[21] J.K.H. Chiu, R.T.H. Ong, "Clustering biological sequences with dynamic sequence similarity threshold", *BMC Bioinformatics* vol. 23, 2022

[22] E. Bao et al. "Seed: efficient clustering of next generation sequences. Bioinformatics", *Bioinformatics*, vol, 27, pp. 2502–2509, 2011

[23] E. Zorita, P. Cuscó, G. J. Filion, "Starcode: sequence clustering based on all-pairs search", *Bioinformatics*, vol. 41, pp. 1913–1919, 2015

[24] M. Ester et al. "A Density-Based Algorithm for Discovering Clusters in Large Spatial Databases with Noise", *AAAI Press*, 1996

[25] G. Qu, Z. Yan, H. Wu., "Clover: tree structure-based efficient DNA clustering for DNA-based data storage", *Briefings in Bioinformatics*, Vol. 23, 2022

[26] T. Shinkar et al. "Clustering-Correcting Codes", *IEEE Transactions on Information Theory*, vol. 68, pp. 1560-1580, 2022

[27] C. Rashtchian et al. "Clustering billions of reads for DNA data storage", *31st NIPS*, pp. 3362–3373, 2017.

[28] P. L. Antkowiak et al. "Low cost DNA data storage using photolithographic synthesis and advanced information reconstruction and error correction", *Nat Commun*, Vol. 11, 2020

[29] j. Leskovec et al., "Mining of Massive Datasets,", *Cambridge University Press, Cambridge*, 2014.

[30] S. Har-Peled et al., "Approximate nearest neighbor: Towards removing the curse of dimensionality,", *Theory of Computing Exchange*, vol. 8, pp. 321–350, 2012.

[31] G.Marçais et al., "Locality-sensitive hashing for the edit distance,", *TBioinformatics*, vol. 35, pp. 127–135, 2019

[32] J. Buhler, "Efficient large-scale sequence comparison by locality-sensitive hashing,", *TBioinformatics*, vol. 17, pp. 419–428, 2001

[33] A. Carass et al., "Evaluating White Matter Lesion Segmentations with Refined Sørensen-Dice Analysis", *Scientific Reports*, 2020.

[34] S. Sankar et al., "Comparative Analysis of Clustering Methodologies in DNA Storage", *26th ICSEC* pp. 269-274, 2022.

[35] G. Chaykin et al. "DNA-Storalator: End-to-End DNA Storage Simulator", *13th Non-Volatile Memories Workshop*, 2022.

[36] O, Sabary et al. "SOLQC: Synthetic Oligo Library Quality Control tool", *Bioinformatics*, vol. 37, pp. 720–722, 2021.
